# Community-onset sepsis and its public health burden: a systematic review

**DOI:** 10.1186/s13643-016-0243-3

**Published:** 2016-05-18

**Authors:** Alexander Tsertsvadze, Pam Royle, Farah Seedat, Jennifer Cooper, Rebecca Crosby, Noel McCarthy

**Affiliations:** Communicable Disease Control Epidemiology and Evidence; Populations, Evidence and Technologies, Division of Health Sciences, Warwick Medical School, University of Warwick, Coventry, CV4 7AL UK; Division of Health Sciences, Warwick Medical School, University of Warwick, Coventry, CV4 7AL UK; NIHR Health Protections Research Unit in Gastrointestinal Infections, University of Oxford, Oxford, UK

**Keywords:** Community-onset sepsis, Risk factors, Incidence of sepsis or severe sepsis

## Abstract

**Background:**

Sepsis is a life-threatening condition and major contributor to public health and economic burden in the industrialised world. The difficulties in accurate diagnosis lead to great variability in estimates of sepsis incidence. There has been even greater uncertainty regarding the incidence of and risk factors for community-onset sepsis (COS). We systematically reviewed the recent evidence on the incidence and risk factors of COS in high income countries (North America, Australasia, and North/Western Europe).

**Methods:**

Cohort and case-control studies were eligible for inclusion. Medline and Embase databases were searched from 2002 onwards. References of relevant publications were hand-searched. Two reviewers screened titles/abstracts and full-texts independently. One reviewer extracted data and appraised studies which were cross-checked by independent reviewers. Disagreements were resolved via consensus. Odds ratios (ORs) and 95 percent confidence intervals (95 % CIs) were ascertained by type of sepsis (non-severe, severe, and septic shock).

**Results:**

Ten cohort and 4 case-control studies were included. There was a wide variation in the incidence (# cases per 100,000 per year) of non-severe sepsis (range: 64–514), severe sepsis (range: 40–455), and septic shock (range: 9–31). Heterogeneity precluded statistical pooling. Two cohort and 4 case-control studies reported risk factors for sepsis. In one case-control and one cohort study, older age and diabetes were associated with increased risk of sepsis. The same case-control study showed an excess risk for sepsis in participants with clinical conditions (e.g., immunosuppression, lung disease, and peripheral artery disease). In one cohort study, higher risk of sepsis was associated with being a nursing home resident (OR = 2.60, 95 % CI: 1.20, 5.60) and in the other cohort study with being physically inactive (OR = 1.33, 95 % CI: 1.13, 1.56) and smoking tobacco (OR = 1.85, 95 % CI: 1.54, 2.22). The evidence on sex, ethnicity, statin use, and body mass index as risk factors was inconclusive.

**Conclusions:**

The lack of a valid standard approach for defining sepsis makes it difficult to determine the true incidence of COS. Differences in case ascertainment contribute to the variation in incidence of COS. The evidence on COS is limited in terms of the number and quality of studies. This review highlights the urgent need for an accurate and standard method for identifying sepsis. Future studies need to improve the methodological shortcomings of previous research in terms of case definition, identification, and surveillance practice.

**Systematic review registration:**

PROSPERO CRD42015023484

**Electronic supplementary material:**

The online version of this article (doi:10.1186/s13643-016-0243-3) contains supplementary material, which is available to authorized users.

## Background

### Health and economic burden

Sepsis is a complex life-threatening condition characterised by the host’s systemic inflammatory immune response to infection, which may lead to organ damage, organ failure, septic shock, and death [1]. Sepsis with its associated complications remains a major public health and economic burden in the industrialised world [2]. Outcomes of sepsis may have serious short- or long-term consequences such as amputation, damage to organs, or cognitive dysfunction. In the US, treatment of a patient with sepsis may cost up to $50,000, translating to an annual nationwide economic burden of $17 billion [3, 4]. In European studies, the treatment of severe sepsis in 2002 was estimated to cost approximately £25,000 [5]. Assuming an incidence of 100,000 new cases per year, the UK’s National Health Service (NHS) expenditure for treating these cases would amount to £2.5 billion annually [6].

Estimates of sepsis incidence vary, which may be due to the lack of a uniform definition, disease heterogeneity, and differences in data sources/case ascertainment (e.g., clinical registries, hospital discharge databases, or vital statistics records) [4]. Large nation-wide cohort studies conducted in five high-income countries (the UK, the USA, Australia, France, and New Zealand) [3, 4, 7–9], showed a wide variation in the annual incidence of severe sepsis ranging from 51 [7] to 300 [3] cases per 100,000 population.

Furthermore, the evidence accumulated over the past two decades has shown a gradual increase (8 %-13 % per year) in the incidence of sepsis in high-income countries (the UK, the USA) [4, 10–12]. This trend could be due to the effects of aging populations (e.g., higher proportions of elderly, type-II diabetes, cancer) and improvements in the methods of detection [13, 14].

### Definition and diagnosis

The current definition of sepsis introduced in 1991 [15] and updated in 2001 [1] encompasses the presence of infection and more than one of the Systemic Inflammatory Response Syndrome (SIRS) criteria including: a) body temperature [>38 °C or <36 °C], b) heart rate [>90 beats/min], c) hyper-ventilation [respiratory rate >20 breaths/min or PaCo2 < 32 mmHg], and d) White Blood Cell Count [>12,000 cells/μL or < 4,000 cells/μL]. According to this definition, sepsis with organ dysfunction and sepsis with acute circulatory failure with arterial hypotension have been termed as severe sepsis and septic shock, respectively [1]. The utility of joint infection and SIRS criteria as a diagnostic tool is limited owing to its high sensitivity and low specificity [10, 16]. Some authors have suggested that this definition should additionally incorporate a more specific sign of sepsis that provides evidence of organ dysfunction [13, 14].

The variation in case definition complicates the comparison of findings across studies.

### Community-onset sepsis

Depending on the place of acquisition, sepsis is classified into community-onset and hospital-acquired (i.e., nosocomial) infection [17]. The two contexts of sepsis acquisition differ in the host characteristics (e.g., demographics, risk profile, resistance patterns), pathogens, and outcomes [18–21]. The definition of community-onset sepsis (COS) in the literature has not been consistent [22]. One widely used definition of COS is the presence of positive blood culture and SIRS criteria before or within 48 hours of hospital admission [19, 21, 23–25]. The majority of studies have not attempted to distinguish between COS and hospital-acquired sepsis. This is an important evidence-based gap for planning public facing interventions.

In this systematic review, we aimed to synthesise the recent evidence on the incidence and risk factors of COS in the western industrialised world (North America, Australasia, and North/Western Europe). This independent review was undertaken to provide evidence for Public Health England (PHE). Motivation for estimating the overall burden and risk factors of COS was to contribute information for community-based interventions to guide patients and the public in health-care seeking behaviour.

## Methods

This review was conducted based on the previously published protocol [26]. The modification of quality assessment strategy was the only deviation from the methodology described in the protocol. This review is reported according to the recommendations from the Preferred Reporting Items for Systematic Review and Meta-Analysis (PRISMA) statement. The corresponding PRISMA checklist is provided as an Additional file 1 [27].

The review focused on the evidence from countries located in North America, Australasia, and North/Western Europe. Given the longitudinal changes in the incidence, modifications in the definition [10] and introduction of ICD-9 coding of sepsis [28], we restricted our focus to studies providing data that had been collected in 2002 or onwards.

### Study inclusion criteria

English-language full-text reports of cohort and case-control studies reporting incidence of and/or risk factors for COS were included.

- *Study setting*: population- or hospital-based studies considering COS separately
- *Population*: community dwellers, hospitalised patients (male/female) of any age (except for neonates) from a defined population with or without COS at study baseline. The study used relevant ICD-9/10 codes [3, 4] and established criteria for the diagnosis of sepsis [1].
- *Exposure/risk factor*: any patient characteristic or clinical parameter (e.g., age, sex, co-morbidity, heart rate, body temperature) associated with a risk of COS
- *Outcome*: annual incidence (per population/hospital admission) of COS; risk factors for COS.

We excluded studies of nosocomial sepsis and those unable to differentiate COS; intervention and prognostic studies; studies reporting only single-site infection or single infecting species; and studies limited to specific subgroups (e.g., neonates) or clinical conditions (e.g., cancer, coronary heart disease).

### Search strategy

We searched Medline and Embase from 01 January, 2002 to May 8, 2015, using a combination of subject headings and keywords for sepsis and related terms combined with terms for epidemiology and community based settings.

We searched for unpublished literature through the following sources: a) hand search of reference lists, b) relevant websites of organizations dealing with sepsis (International Sepsis Forum, Sepsis Trust UK, Sepsis Alliance, Centre for Disease Control, World Sepsis Day), c) contacting experts in the field, d) theses database (index to theses), and e) Google Scholar (government or other reports). We did not search abstracts from conference proceedings, since they do not have full texts and therefore do not provide sufficient information to verify a) how sepsis was diagnosed, b) whether study population had COS or other sepsis, and c) details on incidence and risk factors. The Medline and Embase search strategies are provided in Additional file 2.

### Study selection

All bibliographic records identified were compiled and de-duplicated in an endnote database. Two independent reviewers (AT and FS) using a pre-defined piloted form screened all titles/abstracts and later full text reports of potentially eligible records. Disagreements at both levels were resolved via consensus.

### Data extraction

One reviewer (AT) used a pre-piloted sheet and extracted relevant information before it was checked by two independent reviewers (JC and RC). Disagreements between the reviewers were resolved via consensus. The extracted data included information on study (e.g., author name, year of publication, country of conduct, design, study setting, sample size), risk factors (e.g., socio-demographic characteristics, co-morbidities, clinical symptom or parameter), and outcomes (e.g., definition of sepsis, type of pathogen, incidence). Missing statistical parameters of importance were calculated, if data permitted. The data extraction sheet is provided in Additional file 3.

### Quality assessment

Methodological quality of studies was appraised by one reviewer (AT) and checked independently by another reviewer (JC). The assessments were done using two checklists by the Scottish Intercollegiate Guidelines Network (SIGN) for cohort [29] and case-control studies [30]. We selected these tools based on the guidance for evidence-based decision making in infectious diseases epidemiology, prevention, and control proposed by Harder and colleagues [31].

Both checklists address five sources of bias: study research question, participant selection, information bias, confounding, statistical analysis, and an overall assessment of the study (i.e., summary judgement on internal and external validity). The overall quality ratings (high, acceptable, or low) were based on the extent to which the pre-selected important domains of bias were affected in cohort (Selection of subjects: items 4–5; Assessment: items 7, 10–11; Confounding: item 13) and case-control studies (Selection of subjects: items 3–4, 6–7; Assessment: item 9; Confounding: item 10). The checklists and assessments are provided in Additional file 4.

### Data analysis and synthesis

The evidence was synthesized and organised in summary tables and text. The incidence and risk factor data were stratified by study design and sepsis severity: non-severe, severe, and septic shock. The overall sepsis incidence was expressed as the annual number of new cases per population, cumulative incidence proportion (CIP; in %) or incidence density rate (IDR). The association between risk factors and sepsis was expressed with odds ratios (ORs) and hazard rate ratios (HRRs) with 95 percent confidence intervals (95 % CIs).

Meta-analysis could not be performed due to extensive heterogeneity across study population characteristics, exposure definitions, and the outcome measurement methodology. The scarcity of evidence did not permit to determine the effects of publication bias and the conduct of sub-group analysis by age, sex, study setting, and place of acquisition (community-acquired vs. healthcare-associated community onset).

## Results

### Literature search and included studies

All the searches (electronic databases, hand search, contacting authors, and auto alerts) identified 6,351 bibliographic records. No additional records were found through the websites of sepsis organizations, theses database, and Google Scholar.

**Fig. 1:**
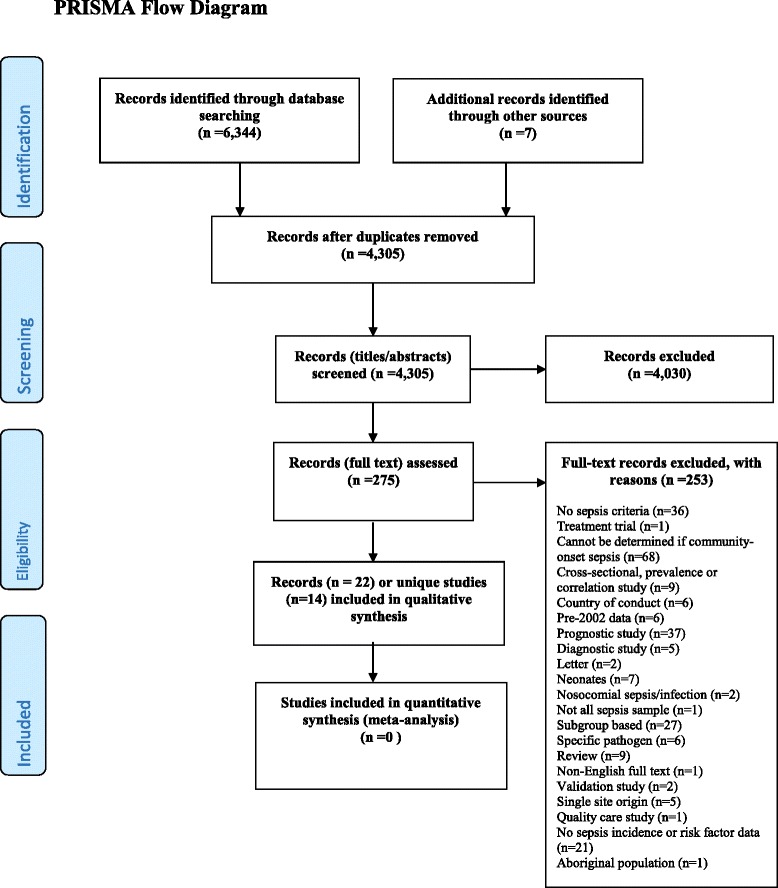
PRISMA Flow Diagram

After duplicates were removed, a total of 4,305 titles/abstracts were screened, of which 275 passed to full text screening level. Of the 275 full text reports examined, only 22 met the inclusion criteria (representing 14 studies) [11, 32–52]. The study selection process and reasons for exclusion at the full-text screening level are presented in Fig. 1 (the PRISMA Flow Diagram) [27].

Of the 14 included studies, 10 were cohort studies [11, 32–38, 40–42, 46–52] and 4 case-control studies [39, 43–45].

One cohort study, which was supported by 9 publications [32–38, 40, 41], used the data from the Reasons for Geographic And Racial Differences in Stroke (REGARDS) study database [53]. In this review, this cohort study is referred as the REGARDS-sepsis cohort study and as a whole it is referenced as Wang et al. 2012 [36]. Two case-control studies by Wang et al. [39] and Henriksen et al. [43] were based on the data from the REGARDS-sepsis study [36] and the cohort study by Henriksen et al. [42], respectively. Although based on the same cohort data, these two case-control studies have been treated as distinct entities from their cohort study counterparts in the data synthesis because of the different design and outcomes reported (i.e., no overlap or double-counting of studies per outcome). The Wang et al. case-control study [39] is referred to as the REGARDS-sepsis case-control study.

### Study and population characteristics

Study population characteristics for the included studies are provided in Table 1 (cohort studies) and Table 2 (case-control studies).

**Table 1:** Study and population characteristics: cohort studies

Study ID year [country]	Study characteristics	Population characteristics	Case identification and data source	Exposure and follow-up
Esteban 2007 [46] Spain	**Design:** prospective	**Case definition and criteria used:** ACCP/SCCM definitions ^μ^	Case report forms	**Exposure:** Not defined
	**Study setting:** ICU, hospital ward	**Criteria for COS:** 83% sepsis patients had a community-acquired infection (no other details given)		**FU:** March 1, 2003 to June 30, 2003
	**Geographic scope:** city/municipal	**Inclusion criteria:** Consecutively admitted patients (age≥18 years)		**FU duration**: 4 months
	**Recruitment years:** March 1, 2003 to June 30, 2003	**Description (n sample size):** 15,852 (includes n=702 non-severe sepsis cases)		
Ginde 2013 [47] The USA	**Design:** retrospective	**Case definition and criteria used:** Severe sepsis as concurrent bacterial or fungal infection plus acute organ dysfunction using ICD-9-CM codes (995.92) for infection and acute organ dysfunction	Annual survey of ED visits conducted by the National Centre for Health Statistics (associated with U.S. non-institutional, general and short stay hospitals)	**Exposure:** Age, nursing home residence
	**Study setting:** nursing residence, community-living, ED			
				**FU:** 2005 to 2009
		**Criteria for COS:** patients admitted to ED for severe sepsis		**FU duration**: 5 years
	**Geographic scope:** Nationwide	**Inclusion criteria:** adults (≥18 years) who visited ED (with or without infection other than sepsis)		
	**Recruitment years:** 2005–2009	**Description (n sample size**): a nationally representative survey of all ED visits of adults (age ≥ 18 years) in 2005–2009 (n=87,500,000)		
Harrison 2006 [11] The UK	**Design:** retrospective	**Case definition and criteria used:** severe sepsis - infection plus ≥3 SIRS criteria and ≥1 organ dysfunction during the 24-hour period	The Case Mix Programme Database containing data on demographics, case mix, outcome and activity for admissions. Patient data were abstracted by trained data collectors according to precise rules and definitions	**Exposure:** not defined
	**Study setting:** ICU and combined ICU/HDU			**FU:** December 2001 to January 2005
		**Criteria for COS:** as having severe sepsis during the first 24 hours following admission to the critical care unit		
	**Geographic scope:** Nationwide (England, Wales, and Northern Ireland)			**FU duration**: 2 years (2002-2004)
		**Inclusion criteria:** adults (≥16 years) presenting with severe sepsis within 24 hours of admission to critical care		
		**Description (n sample size):** n=59,388 (2002), n=59,527 (2003), n=24,905 (2004)		
	**Recruitment years:** December 1995 to January 2005			
Henriksen 2015a [42] Denmark	**Design:** Prospective	**Case definition and criteria used:** the ACCP/SCCM criteria^μ^	The hospitals’ patient administrative database and electronic patient records which were manually reviewed.	**Exposure:** age, sex
	**Study setting:** medical ED, ICU, community-based	**Criteria for COS:** Patient had an infection at arrival or developed an infection during the first 48 hours after admission.		**FU:** September 1, 2010 to August 31, 2011
				**FU duration**: 12 months
	**Geographic scope:** city/municipal			
		**Inclusion criteria:** adults (≥15 years) admitted to the medical ED or directly to the medical ICU		
		**Description (n sample size):** A population-based survey of all adult		
	**Recruitment years:** September 1, 2010, to August 31, 2011	patients admitted to the medical ED at Odense University Hospital, Denmark (n=235,598); n=8,358 hospitalisations		
Husak 2010 [48] Canada	**Design:** retrospective	**Case definition and criteria used:** Severe sepsis (including septic shock) - sepsis complicated by organ dysfunction in at least one of the six organ systems and the ICD codes	Hospital discharge abstract database	**Exposure:** Not defined
	**Study setting:** hospital ward, ED, ICU			**FU:** April 1, 2004 to March 31, 2009
	**Geographic scope:** Nationwide	**Criteria for COS:** the majority (79%) of patients with sepsis were admitted via the EDs, while 12.4% were admitted directly through hospitals		**FU duration**: 5 years
	**Recruitment years:** April 1, 2004 to March 31, 2009			
		**Inclusion criteria:** NR		
		**Description (n sample size):** sepsis cases n=26,803 (2004-2005), n=30,587 (2008-2009), n for non-cases (all non-sepsis hospitalizations): NR		
Nygard 2014 [49] Norway	**Design:** prospective	**Case definition and criteria used:** ACCP/SCCM^μ^ and SCCM/ESICM/ACCP/ATS/SIS	Clinical data were registered prospectively until hospital discharge or in-hospital death using predefined case report forms. Information was collected from medical records, patient charts, and the intensive care electronic monitoring system	**Exposure:** not defined
	**Study setting:** ED, ICU, HDU, and combined ICU/HDU			**FU:** January 2008 to December 2008
		**Criteria for COS:** if they developed severe sepsis within 24 hours of admission to the primary institution		
				**FU duration**: 12 months
	**Geographic scope:** city/municipal	**Inclusion criteria:** patients > 15 years of age hospitalized due to COS, including patients transferred from affiliated hospitals, if they developed severe sepsis within 24 hours of admission to the primary institution		
	**Recruitment years:** January 2008 - December 2008			
		**Description (n sample size):** total N hospitalisations (NR); n=220 severe sepsis cases; non-cases (n): 350,000 population		
Page 2015 [50] The USA	**Design:** retrospective	**Case definition and criteria used:** severe sepsis was defined using the methodology of Angus et al. identifying hospitalizations with the presence of ICD-9 discharge diagnoses for both a serious infection and organ dysfunction	Hospital discharge data from the UHC representing 300 academic and community hospitals across 42 states. Using medical record review, coders assigned discharge diagnoses for each hospitalization	**Exposure:** not defined
	**Study setting:** NR			**FU:** January 1, 2012 to December 31, 2012
	**Geographic scope:** Nationwide			
				**FU duration**: 12 months
	**Recruitment years:** January to December 2012	**Criteria for COS:** Hospitalizations with an infection present at admission were subdivided into healthcare-associated (admitted from nursing facility, receiving home healthcare, or were on haemodialysis prior to admission), community acquired (from the community). Those discharged without infections at admission were categorized as hospital-acquired		
		**Inclusion criteria:** all hospital discharges from January 1, 2012 to December 31, 2012		
		**Description (n sample size):** n**=**3,355,753 hospitalisations; non-cases n (population)=NR		
Wang 2012 [36] REGARDS-sepsis cohort study The USA	**Design:** prospective	**Case definition and criteria used:** An infection plus ≥ 2 SIRS criteria^μ^	Structured interviews, in-home visits, lab results, monitoring every 6 months, medical and hospital admission records (clinical and lab data)	**Exposure:** anthropometric, socio-demographic, dietary, and life-style factors, chronic conditions, and statin use
	**Study setting:** Community-based, hospital ward, ED	**Criteria for COS:** cohort of community-dwelling individuals. The study focused on individuals presenting to the hospital or ED with community-acquired sepsis		
	**Geographic scope:** Nationwide	**Inclusion criteria:** NR		
		**Description (n sample size):** Community-dwelling ≥45 years old individuals in the US (n=30,239)		
				**FU:** 5 February, 2003 to 31 December, 2012
	**Recruitment years:** 2003-2007			
				**FU duration**: ≥ 7 years
Seymour 2012 [51] The USA	**Design:** retrospective	**Case definition and criteria used:** Hospitalization with severe sepsis using the ICD-9-CM (995.92 and 785.52). **Criteria for COS:** pre-hospital severe sepsis	EMS reports computerized database including dispatch, demographic, clinical, and transport data for each incident. EMS data were linked to hospital discharge records	**Exposure:** not defined
	**Study setting:** pre-hospital EMS			**FU:** 2000 to 2009
				**FU duration**: 10 years
	**Geographic scope:** regional (King County in Washington State)	**Inclusion criteria:** all adult EMS encounters involving non-trauma, non–cardiac arrest patients transported from a scene to a receiving hospital by ground ambulance		
	**Recruitment years:** 2000-2009	**Description (n sample size):** all 2000-2009 EMS encounters in the area of 1.2 million residents (n=407,176)		
Wang 2007 [52] The USA	**Design:** retrospective cohort study	**Case definition and criteria used:** the criteria by ACCP/SCCM^μ^ and SCCM/ESICM/ACCP/ATS/SIS; ICD-9 codes 990.90 and 995.92 as sepsis and severe sepsis, respectively	The study used the 2001–2004 NHAMCS public use data set which is a national sample of ED and outpatient visits at hospitals across the US	**Exposure:** not defined
				**FU:** 2001 to 2004
	**Study setting:** ED			**FU duration**: 4 years
	**Geographic scope:** Nationwide	**Criteria for COS:** patients with severe sepsis presenting to ED		
		**Inclusion criteria:** adults (≥18 years) presenting to EDs during 2001-2004 (with or without severe sepsis)		
	**Recruitment years:** 2001-2004			
		**Description (n sample size):** n=113,123 ED visits (sample-based 4 years); 4-year national estimate n=331,531,000 ED visits; annual national estimate n=82,883,000 ED visits		

*NR* not reported, *COS* community-onset sepsis, *ICU* intensive care unit, *HDU* high dependency unit, *FU* follow-up, *SIRS* systemic inflammatory response syndrome, *REGARDS* Reasons for Geographic And Racial Differences in Stroke, *MI* myocardial infarction, *CAD* coronary artery disease, *DVT* deep vein thrombosis, *ED* emergency department, *AF* atrial fibrillation, *BMI* body mass index, *WC* waist circumference, *PAD* peripheral artery disease, *TV* television, *NHAMCS* National Hospital Ambulatory Medical Care Survey, *ICD-9 CM* International Classification of Disease, Ninth Revision Clinical Modification, *IQR* interquartile range, *ACCP/SCCM* American College of Chest Physicians/Society of Critical Care Medicine, *ESICM* European Society of Intensive Care Medicine, *ATS* American Thoracic Society, *SIS* Surgical Infection Society, *ICD-10-CA* International Statistical Classification of Diseases and Related Health Problems, 10th Revision, Canada, *CCI* Canadian Classification of Health Interventions, *EMS* emergency medical services

^μ^ International Sepsis Definitions Conference [1]

^β^ REGARDS-sepsis cohort study publications [32–38, 40, 41]

**Table 2:** Study and population characteristics: case-control studies

Study ID year [country]	Study characteristics	Population characteristics	Identification and data source
Henriksen 2015b [43] Denmark	**Study setting:** medical ED, ICU, community-based	**Case definition:** sepsis (non-severe), severe sepsis, or septic shock according to the ACCP/SCCM criteria.	Data electronically extracted from the patient’s records and validated by trained data. All admissions were manually reviewed. Predefined risk factors retrieved from several population-based registers
		**Control definition:** all adults (≥15 years) with residence in the hospital catchment-area (N = 235,598) during the study period who had not been hospitalized up to 7 days before the index date.	
	**Geographic scope:** city/municipal		
	**Recruitment years:** September 1, 2010 to August 31, 2011	**Criteria for COS:** Patient had an infection at arrival within the first 48 hours after admission	
		**Inclusion criteria (cases):** All adults (≥15 years) admitted to the medical ICU or ED.	
		**Inclusion criteria (controls):** see controls definition and criteria	
		**Exclusion criteria:** cases with a prior hospitalization up to 7 days before the current admission. Patients transferred from other hospitals, patients residing outside the hospitals catchment-area at the time of admission and patients who were unidentified throughout the entire course of admission	
		**N cases:** 1,713 sepsis of any severity (n=621 non-severe sepsis, n=1,071 severe sepsis, and n=21 septic shock)	
		**N controls:** 227,054	
Jovanovich 2014 [44] The USA	**Study setting:** tertiary-level care centres and small clinics and hospitals	**Case definition:** adults hospitalized for sepsis or severe sepsis; ICD-9 codes (995.91, 995.92)	Electronic health and administrative data
		**Control definition:** randomly selected adult patients without sepsis diagnosis admitted within the same time period and matched 1:1 with cases by age, sex, race, and season of 25(OH)D measurement	
	**Geographic scope:** Inter-State (Utah and Idaho)	**Criteria for COS:** community-living adults	
		**Inclusion criteria (cases):** NR	
		**Inclusion criteria (controls):** NR	
	**Recruitment years:** 1 January 2008 and 31 December 2010	**Exclusion criteria:** NR	
		**N cases:** 211	
		**N controls:** 211	
Legras 2009 [45] France	**Study setting:** ICU, hospital ward	**Case definition:** the ACCP/SCCM criteria was used (severe sepsis or septic shock)	Medical histories and data on previous prescriptions obtained from relatives and general practitioner. NSAID use was quantified by listing all the drugs taken during the observation period, and standard interviews were conducted by physicians
	**Geographic scope:** regional	**Control definition:** Participants admitted to hospital for mild bacterial infection (without severe sepsis or septic shock)	
	**Recruitment years:** February 2004 to November 2005	**Criteria for COS:** Community-acquired (NR)	
		**Inclusion criteria (cases):** Participants >15 years admitted to an ICU with community-onset severe sepsis or septic shock	
		**Inclusion criteria (controls):** Participants admitted to hospital for mild bacterial community-acquired (non-sepsis) infection	
		**Exclusion criteria:** chronic kidney failure (creatinine clearance <30 ml/min), pregnancy, nosocomial infection, or congenital/acquired immunosuppression (defined as the presence of metastatic neoplasia, haemopathy, aplasia before the onset of sepsis), AIDS (independently of CD4+ T-cell count) and chronic administration of immunosuppressive treatments	
		**N cases:** n=152 (n=34 sever sepsis; n=118 septic shock)	
		**N controls:** 152	
Wang 2013c [39] REGARDS-sepsis case-control study The USA	**Study setting:** community-based, hospital, ED	**Case definition:** laboratory confirmed infection plus ≥ 2 SIRS criteria	Structured interviews, in-home visits, lab results, monitoring every 6 months, medical and hospital admission records (clinical and lab data); blood samples collected from fasting subjects at their homes
		**Control definition:** hospitalized for a serious infection (but did not meet sepsis criteria)	
		**Criteria for COS:** cohort of community-dwelling individuals. Presentation to the hospital consisted of the time of Emergency Department triage or admission to inpatient unit (for participants admitted directly to the hospital). To allow for acute changes in the participant's condition during early hospitalization, we used vital signs and laboratory test results for the initial 28 h of hospitalization. Our study focused on individuals presenting to the hospital or ED with community-acquired sepsis. We did not include “hospital-acquired” sepsis developing at later points of hospitalization	
	**Geographic scope:** Nationwide		
	**Recruitment years:** January 2003- October 2007		
		**Inclusion criteria (cases):** Patients hospitalized for sepsis during the observation period were eligible.	
		**Inclusion criteria (controls):** Individuals with serious infection who did not experience a hospitalization for sepsis, matching for age (±5 years), sex, and time epoch	
		**Exclusion criteria:** individuals hospitalized for conditions unrelated to infections	
		**N cases:** 162	
		**N controls:** 162	

*NR* not reported, *COS* community-onset sepsis, *SIRS* systemic inflammatory response syndrome, *ED* emergency department, *ICU* intensive care unit, *ACCP/SCCM* American College of Chest Physicians/Society of Critical Care Medicine, *GI* gastrointestinal, *CVD* cardiovascular disease, *OR* odds ratio, *95% CI* 95 percent confidence interval, *AIDS* acquired immunodeficiency syndrome, *NSAID* non-steroidal anti-inflammatory drugs, *IL-6* interleukin-6, *TNF-α* tumor necrosis factor alpha, *ICAM* intercellular adhesion molecule, *VCAM* vascular cell adhesion molecule, *DVT* deep vein thrombosis, *CKD* chronic kidney disease, *MI* myocardial infarction, *CAD* coronary artery disease

#### Cohort studies

Of the 10 cohort studies, 4 were of prospective [36, 42, 46, 49] and 6 were of retrospective design [11, 47, 48, 50–52].

In most studies, the settings were emergency departments, intensive care units, high dependence units, and hospital wards. The geographic scope of catchment area varied from nationwide [11, 36, 47, 48, 50, 52] to regional [51] or municipal [42, 46, 49]. The length of follow-up ranged from 4 months [46] to 10 years [36, 51].

Studies enrolled cohorts of patients hospitalised, admitted, or visiting ED/ICU for different reasons (e.g., medical, surgery, trauma, or infection) [11, 42, 46–50, 52]. The REGARDS-sepsis cohort study was the only study with a well-defined study-base sample which consisted of community-dwelling people who were sepsis-free at the study baseline [36]. The population in the REGARDS-sepsis cohort study included those aged 45 years or older [36].

The majority of studies used the standard consensus-based criteria for non-severe and severe sepsis as outlined by Levy et al. [1]. Only 5 studies reported definitions of COS explicitly (i.e., sepsis manifested within the first 24–48 hours of admission) [11, 36, 42, 49, 50].

Data sources utilised were administrative databases ranging from clinical records [11, 36, 42, 46, 49, 52] to hospital discharge records [47, 48, 50]. One study used an emergency medical services encounters database linked with hospital discharge records [51].

#### Case-control studies

This review included 4 case-control studies [39, 43–45]. The REGARDS-sepsis case-control study [39], analysed only a subset of 162 case-control matched pairs sampled from the REGARDS-sepsis cohort study. In contrast, Henriksen et al. [43], analysed all sepsis cases (n = 1,713) and controls (n = 227,054; residents not hospitalised up to 7 days before the index date) from the cohort study base [42]. The study by Jovanovich et al. [44] investigated 211 matched case-control pairs admitted to tertiary care centres, small clinics, and hospitals. Legras and colleagues [45], analysed 211 matched case-control (mild bacterial infection) pairs admitted to intensive care units or hospital wards. The sepsis definitions used were based on the standard criteria [1] and ICD-9 codes (995.91, 995.92).

### Quality of included studies

Methodological quality assessments are presented in Additional file 4 (Table S1: cohort studies and Table S2: case-control studies).

#### Cohort studies

Of the 10 cohort studies, only 4 were rated as of acceptable quality [11, 36, 42, 46]. The 6 remaining studies were judged to be of low quality [47–52]. Poor reporting (item response: can’t say) for the absence of outcome at baseline (item 4) and the validity of methods for the outcome assessment (item 11) were main contributory factors for the cohort studies judged to be of low quality. Also, it was not clear how comparable the exposure groups in these studies were. Three cohort studies with acceptable quality were of prospective design and described explicitly defined populations free of sepsis at baseline (i.e., denominators) [36, 42, 46].

#### Case-control studies

All 4 case-control studies were of low quality [39, 43–45]. Specifically, the controls in three studies were cases hospitalised for serious infection (item 7) [39], mild bacterial infection [45], or non-specified medical condition [44]. Only one study utilised true non-cases as controls [43]. It was not clear if the same exclusion criteria was applied to both cases and controls for two studies [43, 44] and how valid the exposure measurement was in two studies [43, 45].

### Review outcomes

#### Incidence of COS

Data on the incidence of COS is provided in Table 3.

**Table 3:** Incidence of community-onset sepsis: cohort studies

Study ID country	Cohort and study characteristics	Study design and duration of follow-up	Type of sepsis	Incidence – overall (total cohort)			Methodological quality (high, acceptable, low)
				N of cases per 100,000 population per year [95% CI]	CIP % per hospitalisation or ED visit per year [95% CI]	IDR (N of cases per 100,000 p-y) [95% CI]	
Esteban 2007 [46] Spain	**Geographic scope**	Prospective cohort study	Non-severe	367 [352, 384]	13.28% [12.76, 13.81]	NR	Acceptable quality
	City/municipal		Severe	104 [96, 113]	3.76% [3.47, 4.06]	NR	
		FU: 4 months					
	**Setting**		Septic shock	31 [27, 36]	1.11% [0.95, 1.28]	NR	
	ICU, hospital ward						
	**Cohort denominator**						
	N (population)=573,149						
	N (hospitalisations)=15,852						
Ginde 2013 [47] The USA	**Geographic scope**	Retrospective cohort study	Severe	NR	0.40% [0.39, 0.41]	NR	Low quality
	Nationwide						
	**Setting**	FU: 5 years					
	ED						
	**Cohort denominator**						
	N (population)=NR						
	N (all ED visits)=87,500,000						
Harrison 2006 [11] The UK	**Geographic scope**	Retrospective cohort study	Severe	66 [NR]^μ^	27.87% [27.52, 28.24]	NR	Acceptable quality
	Nationwide						
	**Setting**	FU: 2 years					
	ICU, combination of ICU with HDU						
	**Cohort denominator**						
	N (population)=NR						
	N (hospitalisations)=59,527						
Henriksen 2015a [42] Denmark	**Geographic scope**	Prospective cohort study	All	727 [693, 762]	NR	731 [697, 767]	Acceptable quality
	City/municipal		Non-severe	264 [243, 285]	NR	265 [245, 287]	
	**Setting**	FU: 1 year	Severe	455 [428, 482]	NR	457 [430, 485]	
	ED, ICU		Septic shock	9 [6, 13]	NR	9 [6, 14]	
	**Cohort denominator**						
	N (population )=235,598						
	N (hospitalisations)=8,358						
Husak 2010 [48] Canada	**Geographic scope**	Retrospective cohort study	All	103 [NR]^μ^	NR	NR	Low quality
	Nationwide		Non-severe	64 [NR]^μ^	NR	NR	
	**Setting**	FU: 5 years	Severe	40 [NR]^μ^	NR	NR	
	ED, ICU, hospital ward						
	**Cohort denominator**						
	N (population or hospitalisations)=NR						
Nygard 2014 [49] Norway	**Geographic scope**	Prospective cohort study	Severe	50 [NR]^μ^	0.22% [NR]^μ^	NR	Low quality
	City/municipal						
	**Setting**	FU: 1 year					
	ED, ICU, HDU, combination of ICU with HDU						
	**Cohort denominator**						
	N (population or hospitalisations)=NR						
Page 2015 [50] The USA	**Geographic scope**	Retrospective cohort study	Severe	NR	CA-SS	NR	Low quality
	Nationwide				5.75% [5.72, 5.77]		
	**Setting**	FU: 1 year			HCA-SS		
	NR				2.37% [2.35, 2.38]		
	**Cohort denominator**						
	N (population)=NR						
	N (hospitalisations)=3,355,753						
Wang 2012 [36] REGARDS-sepsis cohort study 2012-2015^β^ The USA	**Geographic scope**	Prospective cohort study	Non-severe	514 [489, 539]	NA	800 [760, 840]	Acceptable quality
	Nationwide						
	**Setting**	FU: 9-10 years					
	Hospital ward, ED						
	**Cohort denominator**						
	N (population)=30,239						
	N (hospitalisations)=NR						
Seymour 2012 [51]The USA	**Geographic scope**	Retrospective cohort study	Severe	NR	Entire 10-year cohort	NR	Low quality
	Regional (within-State)				3.25% [3.20, 3.31]		
	**Setting**	FU: 10 years			One-year cohort		
	Pre-hospital emergency medical services				4.93% [4.73, 5.13]		
	**Cohort denominator**						
	N (population)= NR						
	N (emergency encounters)=407,176						
Wang 2007 [52] The USA	**Geographic scope**	Retrospective cohort study	Severe	NR	0.69% [0.61, 0.77]	NR	Low quality
	Nationwide						
	**Setting**	FU: 4 years					
	ED						
	**Cohort denominator**						
	N (population)=NR						
	N (ED visits)=82,883,000						

*CIP* cumulative incidence proportion, *IDR* incidence density rate, *HR* hazard rate, *n/N* number, *p-y* person-years, *95% CI* 95 percent confidence interval; *REGARDS* Reasons for Geographic And Racial Differences in Stroke, *FU* follow-up, *ED* emergency department, *NA* not applicable; *NR* not reported, *ICU* intensive care unit, *HDU* high dependence unit, *CA-SS* community-acquired severe sepsis, *HCA-SS* healthcare-acquired severe sepsis

^μ^ 95 % CIs cannot be calculated, due to the lack of denominator reported

^β^ REGARDS-sepsis cohort study publications [32–38, 40, 41]

##### All sepsis

The incidence of all sepsis (per population) was reported in two studies [42, 48]. The study by Henriksen et al. observed 727 sepsis cases (non-severe, severe, and septic shock; 95 % CI: 693, 762) per 100,000 population per year [42]. The incidence of all sepsis (non-severe and severe) in the study by Husak et al. was estimated to be 103 cases (95 % CI was not reported and could not be calculated) per 100,000 population per year [48].

##### Non-severe sepsis

The incidence of non-severe sepsis (per population) reported in 4 studies [36, 42, 46, 48] ranged from 64 (95 % CI not available) [48] to 514 (95 % CI: 489, 539) [36] cases per 100,000 population per year. Of these, two studies by Esteban et al. [46] and Henriksen et al. [42] reported the incidence of 367 (95 % CI: 352, 384) and 264 (95 % CI: 243, 285) cases, respectively per 100,000 population per year.

##### Severe sepsis

The incidence of severe sepsis (per population) was estimated and provided in 5 studies [11, 42, 46, 48, 49].

The reported estimates across 4 studies [11, 46, 48, 49] ranged from 40 (95 % CI not available) [48] to 104 (95 % CI: 96, 113) [46] cases per 100,000 population per year. The cohort study by Henriksen et al. reported an incidence of 455 (95 % CI: 428, 482) cases of severe sepsis per 100,000 population per year [42].

Six studies also reported the incidence of severe sepsis (per hospitalisation or ED visit) which ranged from 0.22 % (95 % CI not available) [49] to 8.12 % (95 % CI: 8.10, 8.15) [50] per hospitalisation/ED visit per year. The incidence of severe sepsis reported in the study by Harrison et al. was 27.87 % (95 % CI: 27.52, 28.24) [11] per hospitalisation. The incidence of community-acquired severe sepsis (per hospitalisation) was higher compared to healthcare-associated severe sepsis (5.75 % vs. 2.37 %) [50].

##### Septic shock

The incidence estimates for septic shock reported in two studies were 31 (95 % CI: 27, 36) [46] and 9 (95 % CI: 6, 13) [42] per 100,000 population per year.

#### Associations between various factors and the occurrence of sepsis

Two cohort [36, 47] and 4 case-control studies [39, 43–45] contributed relevant data. The associations between socio-demographic factors and COS are provided in Table 4.

**Table 4:** Associations between socio-demographic factors and community-onset sepsis: cohort and case-control studies

Study ID country	Geographic scope and setting	Study design Sample size N	Type of sepsis	Risk factor (reference and exposure groups)	Summary measure of association (exposure vs. reference group) 95% CI	Covariates adjusted for	Methodological quality
**Age (years)**							
Ginde 2013 [47] The USA	Nationwide	Retrospective cohort study	Severe	<65	Ref 1.00	sex and race/ethnicity	Low quality
	ED			≥65	OR=1.00 (0.52, 1.90)		
		N (cohort baseline – all ED visits)= 87,500,000					
Wang 2012 [36] REGARDS-sepsis cohort study ^β^2012-2015 The USA	Nationwide	Prospective cohort study	Non-severe	45-54	Ref 1.00	Not adjusted (crude)	Acceptable quality
	Hospital ward, ED			55-64	HRR=1.44 (1.04, 2.00)		
		N (cohort baseline)=30,239		65-74	HRR=2.29 (1.66, 3.16)		
				75≤	HRR=3.87 (2.80, 5.35)		
Henriksen 2015b [43] Denmark	City/municipal	Case-control study	All	15-64	Ref 1.00	Sex, alcoholism- related conditions, comorbidity, and immunosuppression	Low quality
	ED, ICU	N (cases)=1,713		65-84	OR=3.09 (2.75, 3.48)		
		N (controls)=227,054		≥85	OR=6.02 (5.09, 7.12)		
			Non-severe	15-64	Ref 1.00	See above	
				65-84	OR=2.15 (1.78, 2.60)		
				≥85	OR=3.66 (2.74, 4.88)		
			Severe	15-64	Ref 1.00	See above	
				65-84	OR=3.93 (3.39, 4.56)		
				≥85	OR=7.84 (6.38, 9.63)		
**Sex**							
Ginde 2013 [47] The USA	Nationwide	Retrospective cohort study	Severe	Female	Ref 1.00	Age and race/ethnicity	Low quality
	ED			Male	OR=1.13 (0.62, 2.00)		
		N (cohort baseline– all ED visits)= 87,500,000					
Wang 2012 [36] REGARDS-sepsis cohort study ^β^ 2012-2015 The USA	Nationwide Hospital ward, ED	Prospective cohort study analysis	Non-severe	Female	Ref 1.00	Not adjusted (crude)	Acceptable quality
		N (cohort baseline)=30,239		Male	HRR=1.30 (1.15, 1.48)		
Henriksen 2015b [43] Denmark	City/municipal	Case-control study	All	Female	Ref 1.00	Age, alcoholism- related conditions, comorbidity, and immunosuppression	Low quality
	ED, ICU	N (cases)=1,713		Male	OR=1.01 (0.91, 1.11)		
		N (controls)=227,054					
			Non-severe	Female	Ref 1.00	See above	
				Male	OR=0.89 (0.76, 1.05)		
			Severe	Female	Ref 1.00	See above	
				Male	OR=1.07 (0.95, 1.22)		
**Race/ethnicity**							
Ginde 2013 [47] The USA	Nationwide	Retrospective cohort study	Severe	Non-Hispanic White	Ref 1.00	Age and sex	Low quality
				Non-Hispanic Black	OR=1.30 (0.62, 2.60)		
	ED	N (cohort baseline– all ED visits)= 87,500,000		Hispanic	OR=0.63 (0.23, 1.70)		
				Other	OR=2.40 (0.87, 6.50)		
Wang 2012 [36] REGARDS-sepsis cohort study ^β^ 2012-2015 The USA	Nationwide	Prospective cohort study analysis	Non-severe	Black	Ref 1.00	sex, age, geographic region, education level, income, tobacco, alcohol use, baseline chronic medical conditions, biomarkers	Acceptable quality
	Hospital ward, ED	N (cohort baseline)=30,239		White	HRR=1.56 (1.38, 1.75)		
**Education**							
Wang 2012 [36] REGARDS-sepsis cohort study ^β^ 2012-2015 The USA	Nationwide	Prospective cohort study analysis	Non-severe	≥College	Ref 1.00	Not adjusted (crude)	Acceptable quality
	Hospital ward, ED	N (cohort baseline)=30,239		Some college	HRR=1.41 (1.19, 1.67)		
				High school	HRR=1.52 (1.28, 1.80)		
				<High school	HRR=1.88 (1.54, 2.29)		
**Nursing home residence**							
Ginde 2013 [47] The USA	Nationwide	Retrospective cohort study	Severe	No	Ref 1.00	Age, sex and race/ethnicity	Low quality
	ED			Yes	OR=2.60 (1.20, 5.60)		
		N (cohort baseline)= 87,500,000					

*NR* not reported, *ICU* intensive care unit, *HDU* high dependence unit; *ED* emergency department, *95% CI* 95 percent confidence interval, *REGARDS* Reasons for Geographic And Racial Differences in Stroke, *Ref* reference group, *OR* odds ratio, *HRR* hazard rate ratio, *NSAID* non-steroidal anti-inflammatory drug, *CKD* chronic kidney disease, *IL-6* interleukin-6, *TNF-α* tumor necrosis factor alpha, *ICAM* intercellular adhesion molecule, *VCAM* vascular cell adhesion molecule, *PSS* perceived stress scale, *SD* standard deviation, *Q1-4* dietary intake quartile scores

^β^ REGARDS-sepsis cohort study publications [32–38, 40, 41]

##### Age

One prospective cohort study indicated an increased risk of non-severe sepsis with older age (≥75 years vs. 45–54 years; OR _crude_ =3.87, 95 % CI: 2.80, 5.35) [36]. In contrast, the cohort study by Ginde et al., showed no such evidence (≥65 years vs. <65 years; OR _adjusted_ =1.00, 95 % CI: 0.52, 1.90) [47]. One case-control study showed older age (≥85 years vs. 15–64 years) to be associated with an increased risk of all sepsis (OR _adjusted_ =6.02, 95 % CI: 5.09, 7.12), non-severe sepsis (OR _adjusted_ =3.66, 95 % CI: 2.74, 4.88), and severe sepsis (OR _adjusted_ =7.84, 95 % CI: 6.38, 9.63) [43].

##### Sex

One prospective cohort (the REGARDS-sepsis) study found a significantly increased risk of non-severe sepsis in men compared to women (HRR _crude_ =1.30, 95 % CI: 1.15, 1.48) [36]. In the other cohort study, the risk of severe sepsis in men was not significantly different from that in women (OR _adjusted_ =1.13, 95 % CI: 0.62, 2.00) [47]. One case-control study [43] showed no significant difference between men and women in risk for all (OR _adjusted_ =1.01, 95 % CI: 0.91, 1.11) or severe sepsis (OR _adjusted_ =1.07, 95 % CI: 0.95, 1.22).

##### Race/ethnicity

The effect of race was evaluated in two cohort studies. In the first study, the risk of severe sepsis in Black (OR _adjusted_ =1.30, 95 % CI: 0.62, 2.60) or Hispanic (OR _adjusted_ =0.63, 95 % CI: 0.23, 1.70) participants did not significantly differ from the risk in White participants [47]. The second study however showed White participants to be at a significantly higher risk for non-severe sepsis compared to Black participants (HRR _adjusted_ =1.56, 95 % CI: 1.38, 1.75) [36].

##### Education

One cohort study reported the risk of sepsis by levels of education and found that lower levels of education were associated with an increased risk of non-severe sepsis (<high school vs. ≥college; HRR _crude_ = 1.88, 95 % CI: 1.54, 2.29) [36].

##### Nursing home residence

One retrospective cohort study found a significant association between nursing home residence and risk of severe sepsis (residence vs. no residence; OR _adjusted_ =2.60, 95 % CI: 1.20, 5.60) [47].

##### Other factors

Associations for anthropometric measures, life-style, clinical factors, medication use, and serum biomarkers are provided in Additional file 4 (Table S3).

One prospective cohort study showed no evidence of significant association between BMI groups in relation to the risk of non-severe sepsis (≥40 kg/m^2^ vs. <24.9 kg/m^2^; HRR _adjusted_ =1.14, 95 % CI: 0.81, 1.62) [36]. The same study however, showed an increased risk for non-severe sepsis among current (HRR _crude_ = 1.85, 95 % CI: 1.54, 2.22) and past tobacco smokers (HRR _crude_ =1.64, 95 % CI: 1.42, 1.88) compared to never smokers. Moderate alcohol use compared to no alcohol was associated with a reduced risk of non-severe sepsis (HRR _crude_ =0.78, 95 % CI: 0.67, 0.89) [36]. Participants reporting low levels of exercise (i.e., none) were at increased risk of non-severe sepsis compared to those with high levels of exercise (≥4 times per week; HRR _adjusted_ =1.33, 95 % CI: 1.13, 1.56).

One case-control and one cohort study demonstrated that people with diabetes had an increased risk for all sepsis (OR _adjusted_ =1.82, 95 % CI: 1.57, 2.12) [43] and non-severe sepsis (HRR _adjusted_ =1.78, 95 % CI: 1.53, 2.07) [36]. Moreover, these two studies reported significantly elevated risk of sepsis for patients with various clinical conditions or disorders (e.g., immunosuppression, renal, psychotic, gastrointestinal, neurologic, cardiovascular disease, cancer, lung disease, deep vein thrombosis, stroke, atrial fibrillation) [36, 43].

## Discussion

This systematic review summarised evidence on the burden of COS in terms of incidence and risk factors from 14 studies.

### Incidence of COS

#### Major findings

The annual population-based incidence rates (# of cases per 100,000 population), as reported in the cohort studies of low to acceptable quality, varied widely for non-severe sepsis (range: 64–514), severe sepsis (range: 40–455), and septic shock (range: 9–31). These results confirm sepsis as a substantial health problem, but underline the uncertainty in the precise burden of this condition. The variability in estimates could be due to differences in the underlying risk for sepsis, data sources, ICD-9/10 coding practices, sepsis definition criteria, and statistical methods of incidence estimation (e.g., choice of denominators, dealing with incomplete outcome data).

It has been shown that different ICD-9/10 coding practices (e.g., methods by Angus [3], Dombrovskiy [54], and Martin [4]) used for severe sepsis cases alone may lead to variable incidence estimates [28, 55–57]. Also, there has been confusion in distinguishing bacteraemia, septicaemia, and severe sepsis which are distinct clinical conditions [13, 58–60]. The use of different data sources may also have contributed to this variation. The use of discharge diagnoses and different ICD codes have been shown to produce variable estimates with mostly low sensitivity and high specificity for correct identification of sepsis [13]. Similarly, another study estimated that discharge diagnoses had a high specificity (median: 98.5 %) and poor sensitivity (median: 42.4 %) for detecting true cases of sepsis, thereby leading to misclassification and underdiagnosis of sepsis [57]. The study by Wang et al. [18] corroborated these findings on the sample of community-dwelling adults at risk for developing COS and found that the use of discharge databases was highly specific and poorly sensitive for detecting COS (94.6 % and 27.6 %, respectively). Although more resource-intensive, the utilisation of medical charts allows to review physiologic and laboratory measurements which then may be linked to an underlying infectious pathogen with more certainty. Also, discharge databases unlike medical records do not include the necessary information needed to distinguish community-acquired and hospital-acquired forms of sepsis [18].

The processes occurring over time such as population aging, improvement in detection of sepsis, increased use of immunosuppressive therapy, transplantation, and invasive procedures may also explain the observed variation in sepsis incidence [61]. There is also evidence for changes in the coding and definitions used for sepsis over time. Rhee and colleagues assessed longitudinal data for the annual sensitivity and incidence of discharge ICD-9-CM codes for organ dysfunction (severe sepsis) against clinical criteria [60], and found that from 2005 to 2013, the sensitivity of hospital discharge codes for detecting hospitalisations with severe sepsis had gradually increased (i.e., they have become more inclusive), while clinical thresholds used for defining organ dysfunction had decreased (i.e., they have become less restrictive). The authors concluded that these changes may at least partially explain the increased incidence of severe sepsis over time [60, 62]. To further explore if the observed trends in sepsis incidence have been influenced by the choice of principal or secondary diagnosis codes, Walkey and colleagues [63] using the US population-based sample of hospitalisation claims for sepsis and specific sources of infection (e.g., pneumonia, urinary tract infection, bacteraemia), calculated annual age-standardised hospitalisation rates for sepsis or infections in 2003–2009. The study showed increasing incidence trends for both sepsis (used as principal diagnosis) and infection requiring mechanical ventilation, with the former rising at a greater rate.

The between-study variation in the definitions and methods for identifying and separating community-onset from hospital-acquired sepsis may have also resulted in different degrees of misclassification of sepsis cases, thereby leading to additional variation in the incidence of sepsis.

#### Strengths and limitations of the evidence

Currently, there is great uncertainty across the methods of diagnosis and identification of sepsis. Different ICD-9/10 coding practices yield different estimates of true incidence, and there is no consensus as to which method is more valid in correctly identifying sepsis cases.

The incidence of sepsis reported in 10 cohort studies warrants a cautious interpretation. Specifically, 6 studies were of low [47–52], while the remaining 4 studies were of acceptable methodological quality [11, 36, 42, 46].

The findings from three studies of acceptable quality may have limited applicability to the overall UK population. For example, the annual incidence of non-severe sepsis (514 per 100,000) reported in the cohort study by Wang et al. [36] was dominated by high risk populations (e.g., 60 % of the sample was 60 years or older). The study by Henriksen and colleagues [42], reporting the annual incidence of non-severe sepsis (264 cases per 100,000) was based on a single university-based hospital, and the rates observed may not be applicable to a wider general population. Similarly, the study by Esteban and colleagues, which also reported the annual incidence of non-severe sepsis (264 cases per 100,000) covered only one small metropolitan area of Spain and the study cohort had a short follow-up (4 months) [46].

#### Consistency of findings

This review could not identify another systematic review of COS in order to directly assess the consistency of findings. Incidence rates of sepsis (regardless of the place of acquisition) have been reported in several primary studies [3, 4, 7–9, 28, 55, 64] and reviews [2, 10, 13, 58, 59, 65–67].

Most of the reported evidence pertains to the nationwide and regional incidence rates of severe sepsis rather than non-severe sepsis. For example, a recent study which used an administrative data from 20 % of US hospitals compared the incidence rates of severe sepsis using four methods of case ascertainment. Depending on the method used, the incidence of severe sepsis varied from 300 to 1,031 cases per 100,000 population per year. In their cohort study, Karlsson and colleagues reported an incidence of 38 cases of severe sepsis per 100,000 population per year [64]. An earlier study, based on the 1995 US hospital discharge records reported an estimate of 300 cases of severe sepsis per 100,000 US population per year [3]. According to the Martin et al. study, in 2000, the annual incidence of sepsis in the US was estimated to be about 240 cases per 100,000 population [4].

In one recent systematic review [66], the pooled incidence rates restricted to the 2005–2015 years for sepsis and severe sepsis were 437 (95 % CI: 334, 571; τ = 0.38) and 270 (95 % CI: 176, 412; τ = 0.60) per 100,000 population, respectively.

In general, the estimates of sepsis incidence and the corresponding variability observed in this review were consistent with those of other studies and reviews.

### Risk factors of COS

#### Major findings

Overall, the limited amount of evidence from studies of low to acceptable quality suggested a significantly increased risk of sepsis (non-severe or severe) in association with older age, Caucasian race, lower education, greater waist circumference, nursing home residence, tobacco use, physical inactivity, and various chronic clinical conditions. Evidence for sex, body mass index, alcohol use, statin/non-steroidal anti-inflammatory drug use, and selected endothelial inflammation biomarkers was inconsistent or inconclusive.

#### Strengths and limitations of the evidence

The evidence on risk factors for COS was sparse and based mostly on studies of low methodological quality. Therefore, caution should be exercised when interpreting these results until more definitive evidence is available. Several factors such as selection bias, residual confounding, exposure/outcome measurement misclassification, and multiple testing (i.e., type-I error) could have accounted for some of the observed associations. Of the 4 case-control studies, the control series in 3 studies were non-sepsis cases hospitalised for infections or non-specified medical conditions [39, 44, 45]. The controls used in the Henriksen et al. study were true non-cases [43]. The use of hospital controls is prone to selection bias if their exposure distribution is not representative of that in the base population that gave rise to cases [68]. For some studies, it was not clear if the same exclusion criteria had been applied to cases and controls. The use of different exclusion criteria may lead to biased effect estimates for the reported risk factors of sepsis [68].

#### Consistency of findings

The findings of this review on sex and race are not consistent with those of recent studies not restricted to COS. These studies report sex and racial disparities by showing increased risk of sepsis among males (vs. females) and non-Caucasians (vs. Caucasians) [4, 69, 70]. In our review, one study corroborated these findings and indicated that men were at higher risk for sepsis compared to women [36], but two other studies showed no such evidence [43, 47]. Regarding the effect of race, one included in the review study indicated that Caucasians compared to non-Caucasians were at higher risk of sepsis [33, 36] while another showed no significant difference between the two groups [47]. It is not clear whether these discrepancies were due to the distinct associations applicable to COS, differences in the population at risk, or sepsis coding practices across studies.

In agreement with previous research [10, 59], our review also found that older age and the presence of clinical chronic conditions are associated with an increased risk for sepsis. This included clinical conditions that are known to impair the human immune system, making the patient more susceptible to various types of infection, among them sepsis.

### Strengths and limitations of the review

This is the first systematic review of recent evidence on the incidence and risk factors of COS. This review was restricted to the evidence analysed in 2002 or later when specific ICD-9 coding for non-severe sepsis (995.91), severe sepsis (995.92), and septic shock (785.52) were introduced [28]. The authors *a priori* operationalised all methodological steps of this review.

There are several limitations to be acknowledged. First of all, studies that used pre-2002 study data were excluded given the changes in definition/coding practices, dynamic nature of sepsis incidence, and evolution of associated pathogens. Although the restriction by date limits the comprehensiveness of evidence and precision of the review findings, we believe that the pre-2002 evidence would not reflect the current context of sepsis accurately in light of the changes in sepsis epidemiology. Although non-English publications were not included, to the best of our knowledge, we are not aware of any empirical evidence showing the effects of language bias on the incidence of sepsis. We also excluded conference abstracts because they do not provide sufficient information to distinguish COS from other types of sepsis. The sparsity of evidence did not allow to explore the extent of publication bias. And finally, the quality assessment tool for cohort studies may not have been equally applicable to exposure and non-exposure cohort studies. Due to poor reporting of publications and subjective nature of study quality assessment tools in general, some misclassification in quality ratings cannot be ruled out.

### Future research recommendations

Future research recommendations according to the PICOTS (Population, Intervention, Comparator, Outcome, Timing, and Setting) framework along with the limitations in evidence are provided as follows:

#### Population and Setting

##### Limitation(s)

There is little evidence on the burden of COS from nation-wide population-based cohort studies. Most of the included cohort studies were hospital-based, covered limited geographic areas, or recruited high-risk subgroups and three of the four included case-control studies utilised non-sepsis cases (i.e., hospital-based controls) as controls which may have led to underestimated associations between the risk factors and sepsis.

##### Recommendation(s)

More evidence is warranted from well conducted population-based prospective cohort studies with samples representative of any given general population. These studies would ideally consist of well-defined cohorts of participants free of sepsis at baseline. Future case-control studies would ideally include random samples of population-based controls from the same source population which gave rise to sepsis cases.

#### Intervention and Comparator (exposure, risk factor groups)

##### Limitation(s)

Due to poor reporting, for most of the included studies it was not clear if the methods of exposure measurement were valid. Misclassification of exposure status may have biased the observed effect estimates for risk factors of sepsis in any direction. Moreover, the problem of multiple testing for various risk factors may have led to type-I error (i.e., spurious statistically significant results).

##### Recommendation(s)

Future studies should ideally use standard and validated methods of exposure measurement. Better reporting of these methodologies used would facilitate the methodological or risk of bias assessment for researchers and practitioners involved in evidence synthesis.

#### Outcome

##### Limitation(s)

The estimates of COS incidence were highly variable perhaps owing to well documented differences or changes in sepsis definition, diagnosis, and ascertainment practices across studies.

##### Recommendation(s)

The use of accurate and standard methodology for sepsis surveillance and ascertainment would ensure more valid estimation and comparability of sepsis incidence and risk factors across studies.

#### Timing

##### Limitation(s)

About 40 % of the included cohort studies had a follow-up shorter than 2 years. Some of the estimates of incidence may have been subject to seasonal variation or any other extraneous factor.

##### Recommendation(s)

Studies with longer follow-up are needed to improve stability and precision around sepsis incidence estimates

### Policy implications

Improvements in the accuracy and consistency of sepsis definition, diagnostic criteria, and standardisation of methods for ascertainment of sepsis are prerequisites for assessing the public health burden of sepsis reliably in order to adequately inform public facing health campaigns. A robust monitoring system to support evaluation of any future interventions will require the development of unbiased national surveillance. More studies investigating specific causative pathogens of sepsis (e.g., meningococcal sepsis) with the corresponding incidence, and the related risk factors would provide additional evidence needed to inform the policy for public facing health campaigns targeted to specific causes of sepsis. The findings of this review will help to inform recommendations in relation to public facing campaigns targeting timely presentation and diagnosis of sepsis in the community and provide a policy for future public health planning.

## Conclusion

This review found a highly variable annual population-based incidence of non-severe (range: 64–514 per 100,000) and severe COS (range: 40–455 per 100,000), likely due to different definitions and ascertainment methods of sepsis across included studies. Limited evidence identified several risk factors for sepsis (e.g., older age, lower education, presence of clinical conditions, nursing home residence, and lower levels of physical activity).

Timeliness and accuracy of diagnosis of sepsis are both crucial aspects for improving the patient’s outcome. It is hoped that findings of this review will inform recommendations on public facing campaigns to improve timely presentation and diagnosis of sepsis in the community and provide a basis for future research and policy for public health planning.

## Additional files

Additional file 1: PRISMA 2009 Checklist. (DOC 63 kb) Additional file 2: Search Strategy. (DOCX 32 kb) Additional file 3: Data extraction sheet. (DOCX 39 kb) Additional file 4: Additional tables for included studies. (DOCX 131 kb)

## Abbreviations

PHE: Public Health England
ICU: intensive-care unit
ICD-9/10: International Classification of Diseases, 9^th^/10^th^ revision
SIRS: Systemic Inflammatory Response Syndrome
ACCP: American College of Chest Physicians
SCCM: Society of Critical Care Medicine
HIV: Human Immunodeficiency Virus
UK: United Kingdom
USA: United States of America
PRISMA: Preferred Reporting Items for Systematic review and Meta-Analysis
REGARDS: Reasons for Geographic And Racial Differences in Stroke
COS: Community-Onset Sepsis
SIGN: Scottish Intercollegiate Guidelines Network
CIP: Cumulative Incidence Proportion
IDR: Incidence Density Rate
OR: Odds Ratio
HRR: Hazard Rate Ratio
ED: Emergency Department
NSAIDs: Non-Steroidal Anti-Inflammatory Drugs
NA: Not Applicable
NR: Nor Reported
CKD: Chronic Kidney Disease
IL-6: Interleukin-6
TNF-α: Tumour Necrosis Factor Alpha
ICAM: Intercellular Adhesion Molecule
VCAM: Vascular Cell Adhesion Molecule
SD: Standard Deviation
PSS: Perceived Stress Scale
DVT: Deep Vein Thrombosis
CVD: Cardiovascular Disease
MI: Myocardial Infarction
CAD: Coronary Artery Disease
AIDS: Acquired Immunodeficiency Syndrome
GI: Gastrointestinal
