# Supplementary material for: Community-onset sepsis and its public health burden: a systematic review
Source: Syst Rev. 2016 May 18;5:81. doi: 10.1186/s13643-016-0243-3 (PMC4870814; doi:10.1186/s13643-016-0243-3)
Supplement: Additional file 2: — Search Strategy. (DOCX 32 kb) [file 13643_2016_243_MOESM2_ESM.docx]

**Additional file 2 -** **Search Strategy**

**Ovid Medline (01 January, 2002 to May 8, 2015)**

1. exp Sepsis/

2. (sepsis or septic?emi* or bact?eremi* or disseminated candidiasis or fung?emi* or septic shock).tw.

3. 1 or 2

4. ep.fs.

5. incidence/

6. exp risk/

7. exp Population Surveillance/

8. exp epidemiologic studies/

9. exp odds ratio/ or exp risk/

10. exp Socioeconomic Factors/

11. exp Ethnic Groups/

12. (epidemiology or incidence or risk or mortality or burden or odds ratio or prevalence).tw.

13. 4 or 5 or 6 or 7 or 8 or 9 or 10 or 11 or 12

14. 3 and 13

15. ((community or population) adj2 (onset or acqui* or based)).tw.

16. long term care facilit*.tw.

17. (nursing home* or care home*).tw.

18. exp Residential Facilities/

19. (prehospital or pre-hospital).tw.

20. out-of-hospital.tw.

21. (sepsis related hospitalization or sepsis related hospitalisation).tw.

22. (hospitalised with sepsis or hospitalized with sepsis).tw.

23. admitted with sepsis.tw.

24. 15 or 16 or 17 or 18 or 19 or 20 or 21 or 22 or 23

25. 14 and 24

26. limit 25 to (english language and yr="2002 -Current")

27. (comment or letter or editorial).pt.

28. 26 not 27

**Ovid Embase (01 January, 2002 to May 8, 2015)**

1. exp Sepsis/

2. (sepsis or septic?emi* or bact?eremi* or disseminated candidiasis or fung?emi* or septic shock).tw.

3. 1 or 2

4. ep.fs.

5. incidence/

6. exp risk/

7. exp Population Surveillance/

8. exp epidemiologic studies/

9. exp odds ratio/ or exp risk/

10. exp Socioeconomic Factors/

11. exp Ethnic Groups/

12. (epidemiology or incidence or risk or mortality or burden or odds ratio or prevalence).tw.

13. 4 or 5 or 6 or 7 or 8 or 9 or 10 or 11 or 12

14. 3 and 13

15. ((community or population) adj2 (onset or acqui* or based)).tw.

16. long term care facilit*.tw.

17. (nursing home* or care home*).tw.

18. exp Residential Facilities/

19. (prehospital or pre-hospital).tw.

20. out-of-hospital.tw.

21. (sepsis related hospitalization or sepsis related hospitalisation).tw.

22. (hospitalised with sepsis or hospitalized with sepsis).tw.

23. admitted with sepsis.tw.

24. 15 or 16 or 17 or 18 or 19 or 20 or 21 or 22 or 23

25. 14 and 24

26. limit 25 to (english language and yr="2002 -Current")

27. (comment or letter or editorial or conference).pt.

28. 26 not 27
