# Supplementary material for: Community-onset sepsis and its public health burden: a systematic review
Source: Syst Rev. 2016 May 18;5:81. doi: 10.1186/s13643-016-0243-3 (PMC4870814; doi:10.1186/s13643-016-0243-3)
Supplement: Additional file 3: — Data extraction sheet. (DOCX 39 kb) [file 13643_2016_243_MOESM3_ESM.docx]

**Additional file 3 –** **Data extraction sheet**

| **Study details** | | | |
| --- | --- | --- | --- |
| **Name of first reviewer:**  **Name of second reviewer:** | | | |
| **First** **author** **surname year of publication**:  **Country**:  **Study** **design**:  **Study** **setting** (e.g., hospital, hospital-ward, intensive care unit, nursing home):  **Geographic scope:**  **Criteria for determining community-onset sepsis:**  **Total** **length** **of** **follow** **up (if applicable)**:  **Cohort name:**  **Funding** (government/private/manufacturer/other - specify): | | | |
| **Aim of the study** | | | |
|  | | | |
| **Cohort study** | | | |
| **Participants – total sample** | | | |
| **Recruitment dates:**  **Inclusion** **criteria**:  **Exclusion** **criteria**:  **Sample size at baseline (n total):**  **Sample size (analyzed n):**  **Follow-up start - end dates:**  **Identification methods/data sources:**  **Lost to follow-up/withdrawals (n):**  **Diagnostic criteria for sepsis:**  **Type of sepsis outcome (e.g., sepsis, severe sepsis, septic shock):**  **Co-morbidity (n [%]):**  **Mean (range or SD) age (years):**  **Women (n [%]):**  **Race/ethnicity (n [%]):**  **Other baseline factors:** | | | |
| **General description of the sample:** | | | |
| **Distribution of pathogens amongst sepsis cases n (%)** | | | |
| **Distribution of infection site/origin/focus n (%)** | | | |
| **Definition of exposure groups (if applicable)**  **Exposure-1 (specify)**  Non-exposed group [n/N]:  Exposed group-1 [n/N]:  Exposed group -2 [n/N]:  **Exposure-2 (specify)**  Non-exposed group [n/N]:  Exposed group-1 [n/N]:  Exposed group -2 [n/N]:  **Exposure-3 (specify)**  Non-exposed group [n/N]:  Exposed group-1 [n/N]:  Exposed group -2 [n/N]:  __________________________________________________________________________________  n= number of new sepsis cases  N=number of participants at baseline or number of person-years | | | |
| **Reported outcomes (i.e., incidence, risk factors):** | | | |
| **Outcome - 1: frequency measures** | | | |
| **SPECIFY (sepsis, severe sepsis, or septic shock)** | **CIP**  **(n/N [%])**  **95% CI** | **IR**  **(n per N person-years)**  **95% CI** | **HR**  **95% CI** |
| Total cohort |  |  |  |
| Exposed group-1  (specify, if applicable) |  |  |  |
| Exposed group-2  (specify, if applicable) |  |  |  |
| Exposed group-3  (specify, if applicable) |  |  |  |

| **Outcome – 2: measures of association** | | | | | | | | | |
| --- | --- | --- | --- | --- | --- | --- | --- | --- | --- |
| **SPECIFY (sepsis, severe sepsis, or septic shock)** | | **Reference population** | | **CIR, IRR, or HRR (specify)**  **95% CI** | | | | **Covariates adjusted for** | |
|  |  |  |  | **Crude** | | **Adjusted** | |  |  |
| Exposure (risk factor) -1  (specify) | |  | |  | |  | |  | |
| Exposure (risk factor) -2  (specify) | |  | |  | |  | |  | |
| Exposure (risk factor) -3  (specify) | |  | |  | |  | |  | |
| **Case-control study** | | | | | | | | | |
| **Participants – total sample** | | | | | | | | | |
| **Recruitment dates**:  **Inclusion** **criteria**:  **Exclusion** **criteria**:  **Cases (n total):**  **Controls (n total):**  **Identification methods/data sources:**  **Diagnostic criteria for sepsis:**  **Type of sepsis outcome (e.g., sepsis, severe sepsis, septic shock):**  **Co-morbidity (n [%]):**  **Mean (range or SD) age (years):**  **Women (n [%]):**  **Race/ethnicity (n [%]):**  **Other baseline factors:** | | | | | | | | | |
| **Distribution of pathogens amongst sepsis cases n (%)** | | | | | | | | | |
| **Distribution of infection site/origin/focus n (%)** | | | | | | | | | |
| **Participants characteristics and outcomes (risk factors of sepsis)** | | | | | | | | | |
| **Type and definition of cases and controls**  **Cases [n]:**  **Controls [n]:** | | | | | | | | | |
| **Risk factors** | **Cases (n=)** | | **Controls (n=)** | | **Association measure**  **(OR and 95% CI)** | | | | |
|  |  |  |  |  | **Crude** | | **Adjusted** | | **Covariates adjusted for** |
| **Risk factor-1** |  | |  | |  | |  | |  |
| **Risk factor-2** |  | |  | |  | |  | |  |
| **Risk factor-3** |  | |  | |  | |  | |  |
| **Risk factor-4** |  | |  | |  | |  | |  |
| **Reviewer’s conclusion** | | | | | | | | | |
|  | | | | | | | | | |
| **Abbreviations** | | | | | | | | | |
