# Supplementary material for: Community-onset sepsis and its public health burden: a systematic review
Source: Syst Rev. 2016 May 18;5:81. doi: 10.1186/s13643-016-0243-3 (PMC4870814; doi:10.1186/s13643-016-0243-3)
Supplement: Additional file 4: — Additional tables for included studies. (DOCX 131 kb) [file 13643_2016_243_MOESM4_ESM.docx]

**Additional file 4 –** **Additional tables for included studies**

**Table S1. Methodological quality of cohort studies (16 items)**

| **Definition (Item #)** | Esteban 2007[[46](#_ENREF_46)] | Ginde 2013 [[47](#_ENREF_47)] | Harrison 2006[[11](#_ENREF_11)] | Henriksen 2015a[[42](#_ENREF_42)] | Husak 2010 [[48](#_ENREF_48)] | Nygard 2014[[49](#_ENREF_49)] | Page 2015 [[50](#_ENREF_50)] | Wang 2012 [[36](#_ENREF_36)]  REGARDS-sepsis cohort study 2012-2015^β^ | Seymour [[51](#_ENREF_51)] | Wang 2007 [[52](#_ENREF_52)] |
| --- | --- | --- | --- | --- | --- | --- | --- | --- | --- | --- |
| **Internal validity** | | | | | | | | | | |
| The study addresses an appropriate and clearly focused question (Item 1) | Yes | Yes | Yes | Yes | No | Yes | Yes | Yes | Yes | Yes |
| **Selection of subjects** | | | | | | | | | | |
| The two groups being studied are selected from source populations that are comparable in all respects other than the factor under investigation (Item 2) | NA | Can’t say | NA | Can’t say | NA | NA | NA | Can’t say | NA | NA |
| The study indicates how many of the people asked to take part did so, in each of the groups being studied (Item 3) | Can’t say | Can’t say | Can’t say | Can’t say | Can’t say | Can’t say | Can’t say | Can’t say | Can’t say | Can’t say |
| The likelihood that some eligible subjects might have the outcome at the time of enrolment is assessed and taken into account in the analysis (Item 4) | Can’t say | Can’t say | Can’t say | Yes | Can’t say | Yes | Yes | Yes | Can’t say | Can’t say |
| What percentage of individuals or clusters recruited into each arm of the study dropped out before the study was completed (Item 5) | NA | Can’t say | NA | Can’t say | NA | NA | NA | Yes | NA | NA |
| Comparison is made between full participants and those lost to follow up, by exposure status (Item 6) | NA | No | NA | No | NA | NA | NA | Yes | NA | NA |
| **Assessment** | | | | | | | | | | |
| The outcomes are clearly defined (Item 7) | Yes | Yes | Yes | Yes | Yes | Yes | Yes | Yes | Yes | Yes |
| The assessment of outcome is made blind to exposure status. If the study is retrospective this may not be applicable (Item 8) | NA | NA | NA | Yes | NA | NA | NA | Can’t say | NA | NA |
| Where blinding was not possible, there is some recognition that knowledge of exposure status could have influenced the assessment of outcome (Item 9) | NA | NA | NA | Yes | NA | NA | NA | Yes | NA | NA |
| The method of assessment of exposure is reliable (Item 10) | NA | Yes | NA | Yes | NA | NA | NA | Yes | NA | NA |
| Evidence from other sources is used to demonstrate that the method of outcome assessment is valid and reliable (Item 11) | Yes | No | Yes | Yes | No | Yes | No | Yes | No | No |
| Exposure level or prognostic factor is assessed more than once (Item 12) | NA | Can’t say | NA | No | NA | NA | NA | No | NA | NA |
| **Confounding** | | | | | | | | | | |
| The main potential confounders are identified and taken into account in the design and analysis (Item 13) | NA | Yes | NA | Yes | NA | NA | NA | Yes | NA | NA |
| **Statistical analysis** | | | | | | | | | | |
| Have confidence intervals been provided? (Item 14) | Yes | Yes | No | Yes | No | No | No | Yes | Yes | Yes |
| **Overall assessment of the study** | | | | | | | | | | |
| Taking into account clinical considerations, your evaluation of the methodology used, and the statistical power of the study, do you think there is clear evidence of an association between exposure and outcome? (Item 15) | NA | No | NA | Yes | NA | NA | NA | Yes | NA | NA |
| Are the results of this study directly applicable to the patient group targeted in this guideline? (Item 16) | No | No | Yes | No | Can’t say | No | No | No | No | No |
| **Summary quality (risk of bias) rating** | Acceptable quality | Low quality | Acceptable quality | Acceptable quality | Low quality | Low quality | Low quality | Acceptable quality | Low quality | Low quality |
| NA=not applicable | | | | | | | | | | |

Possible responses to each item: yes, no, can’t say, or doesn’t apply

High quality (little or no risk of bias; results unlikely to be changed by further research)

Acceptable quality (most criteria met; some flaws in the study with an associated risk of bias; conclusions may change in the light of further studies)

Low quality (either most criteria not met, or significant flaws relating to key aspects of study design; conclusions likely to change in the light of further studies)

^β^ REGARDS-sepsis cohort study publications[[36](#_ENREF_36), [32-35](#_ENREF_32), [37](#_ENREF_37), [38](#_ENREF_38), [40](#_ENREF_40), [41](#_ENREF_41)]

The overall methodological quality of each study was based on the extent to which the pre-selected important domains of bias were affected (response ‘no’ or ‘can’t say’). For cohort studies, these items were the following by each domain of bias:

- Selection of subjects (items 4-5)
- Assessment (item 7, items 10-11)
- Confounding (item 13)

**Table S2. Methodological quality of case-control studies (13 items)**

| **Definition (Item #)** | Henriksen 2015b[[43](#_ENREF_43)] | Jovanovich 2014 [[44](#_ENREF_44)] | Legras 2009 [[45](#_ENREF_45)] | Wang 2013c[[39](#_ENREF_39)]  REGARDS-sepsis  case-control study |
| --- | --- | --- | --- | --- |
| **Internal validity** | | | | |
| The study addresses an appropriate and clearly focused question (Item 1) | Yes | Yes | Yes | Yes |
| **Selection of subjects** | | | | |
| The cases and controls are taken from comparable populations (Item 2) | Yes | Can’t say | Yes | Yes |
| The same exclusion criteria are used for both cases and controls (Item 3) | Can’t say | Can’t say | Yes | Yes |
| What percentage of each group (cases and controls) participated in the study? (Item 4) | Can’t say | No | Can’t say | Can’t say |
| Comparison is made between participants and non-participants to establish their similarities or differences (Item 5) | No | No | No | No |
| Cases are clearly defined and differentiated from controls (Item 6) | Yes | Yes | Yes | Yes |
| It is clearly established that controls are non-cases (Item 7) | Yes | No | No | No |
| **Assessment** | | | | |
| Measures will have been taken to prevent knowledge of primary exposure influencing case ascertainment (Item 8) | Can’t say | Can’t say | No | Yes |
| Exposure status is measured in a standard, valid and reliable way (Item 9) | Can’t say | Yes | Can’t say | Yes |
| **Confounding** | | | | |
| The main potential confounders are identified and taken into account in the design and analysis (Item 10) | Yes | Yes | No | Yes |
| **Statistical analysis** | | | | |
| Confidence intervals are provided (Item 11) | Yes | Yes | Yes | Yes |
| **Overall assessment of the study** | | | | |
| Taking into account clinical considerations, your evaluation of the methodology used, and the statistical power of the study, do you think there is clear evidence of an association between exposure and outcome? (Item 12) | Yes | No | No | Yes |
| Are the results of this study directly applicable to the patient group targeted by this guideline? (Item 13) | No | No | No | No |
| **Summary quality (risk of bias) rating** | Low quality | Low quality | Low quality | Low quality |

Possible responses to each item: yes, no, can’t say, or doesn’t apply

High quality (little or no risk of bias; results unlikely to be changed by further research)

Acceptable quality (most criteria met; some flaws in the study with an associated risk of bias; conclusions may change in the light of further studies)

Low quality (either most criteria not met, or significant flaws relating to key aspects of study design; conclusions likely to change in the light of further studies)

The overall methodological quality of each study was based on the extent to which the pre-selected important domains of bias were affected (response ‘no’ or ‘can’t say’). For case-control studies, these items were the following by each domain of bias:

- Selection of subjects (items 3-4, items 6-7)
- Assessment (item 9)
- Confounding (item 10)

**Table S3. Associations between putative risk factors and community-onset sepsis: cohort and case-control studies**

| **Study ID country** | **Geographic scope and setting** | **Study design**  **Sample size N** | **Type of sepsis** | **Risk factor**  **(reference and exposure groups)** | **Summary measure of association (exposure vs. reference group)**  **95% CI** | **Covariates adjusted for** | **Methodological quality** |
| --- | --- | --- | --- | --- | --- | --- | --- |
| **Body mass index (kg/m^2^)** | | | | | | | |
| Wang 2012[[36](#_ENREF_36)]  REGARDS-sepsis cohort study ^β^  2012-2015  The USA | Nationwide  Hospital ward, ED | Prospective cohort study analysis  N (cohort baseline)=30,239 | Non-severe | 18.5-24.9  <18.5  25.0-29.9  30.0-39.9  ≥40.0 | Ref 1.00  HRR=1.56 (0.84, 2.88)  HRR=0.87 (0.71, 1.08)  HRR=0.81 (0.62, 1.06)  HRR=1.14 (0.81, 1.62) | Age, race, sex, geographic region, income, education, smoking status, alcohol use, exercise, comorbidity | Acceptable quality |
| **Waist circumference (cm)** | | | | | | | |
| Wang 2012[[36](#_ENREF_36)]  REGARDS-sepsis cohort study ^β^  2012-2015  The USA | Nationwide  Hospital ward, ED | Prospective cohort study analysis  N (cohort baseline)=30,239 | Non-severe | ≤102 male/≤88 female  >102cm male/>88cm female | Ref 1.00  HRR=1.47 (1.20, 1.79) | Age, body mass index, race, sex, geographic region, income, education, smoking status, alcohol use, exercise, comorbidity | Acceptable quality |
| **Tobacco use** | | | | | | | |
| Wang 2012[[36](#_ENREF_36)]  REGARDS-sepsis cohort study ^β^  2012-2015  The USA | Nationwide  Hospital ward, ED | Prospective cohort study analysis  N (cohort baseline)=30,239 | Non-severe | Never use  Past use  Current use | Ref 1.00  HRR=1.64 (1.42, 1.88)  HRR=1.85 (1.54, 2.22) | Not adjusted (crude) | Acceptable quality |
| **Alcohol use** | | | | | | | |
| Wang 2012[[36](#_ENREF_36)]  REGARDS-sepsis cohort study ^β^  2012-2015  The USA | Nationwide  Hospital ward, ED | Prospective cohort study analysis  N (cohort baseline)=30,239 | Non-severe | None  Moderate  Heavy | Ref 1.00  HRR=0.78 (0.67, 0.89)  HRR=0.89 (0.64, 1.23) | Not adjusted (crude) | Acceptable quality |
| **Alcoholism-related conditions** | | | | | | | |
| Henriksen  2015b[[43](#_ENREF_43)]  Denmark | City/municipal  ED, ICU | Case-control study  N (cases)=1,713  N (controls)=227,054 | All | No  Yes | Ref 1.00  OR=2.90 (2.41, 3.50) | Age, sex, and comorbidity | Low quality |
|  |  |  | Non-severe | No  Yes | Ref 1.00  OR=2.64 (1.94, 3.59) |  |  |
|  |  |  | Severe | No  Yes | Ref 1.00  OR=2.93 (2.34, 3.67) |  |  |
| **Exercise level** | | | | | | | |
| Wang 2012[[36](#_ENREF_36)]  REGARDS-sepsis cohort study ^β^  2012-2015  The USA | Nationwide  Hospital ward, ED | Prospective cohort study analysis  N (cohort baseline)=30,239 | Non-severe | High  (≥4 times/week)  Medium  (1-3 times/week)  Low (none) | Ref 1.00  HRR=1.02 (0.86, 1.20)  HRR=1.33 (1.13, 1.56) | Age, sex, race, body mass index, geographic region, income, education, smoking status, alcohol use, exercise, comorbidity | Acceptable quality |
| **Television watching** | | | | | | | |
| Wang 2012[[36](#_ENREF_36)]  REGARDS-sepsis cohort study ^β^  2012-2015  The USA | Nationwide  Hospital ward, ED | Prospective cohort study analysis  N (cohort baseline)=30,239 | Non-severe | Low (≤1 hour/day)  Medium (2-3 hour/day)  High (≥4 hour/day) | Ref 1.00  HRR=0.94 (0.79, 1.13)  HRR=1.16 (0.95, 1.40) | Age, race, body mass index, race, sex, geographic region, income, education, smoking status, alcohol use, exercise, comorbidity | Acceptable quality |
| **Stress level (4-item PSS score; range: 0-16)** | | | | | | | |
| Wang 2012[[36](#_ENREF_36)]  REGARDS-sepsis cohort study ^β^  2012-2015  The USA | Nationwide  Hospital ward, ED | Prospective cohort study analysis  N (cohort baseline)=30,239 | Non-severe | 1 SD increase in score | Ref 1.00  HRR=1.04 (0.98, 1.11) | Age, race, body mass index, race, sex, geographic region, income, education, smoking status, alcohol use, exercise, comorbidity | Acceptable quality |
| **Dietary patterns** | | | | | | | |
| Wang 2012[[36](#_ENREF_36)]  REGARDS-sepsis cohort study ^β^  2012-2015  The USA | Nationwide  Hospital ward, ED | Prospective cohort study analysis  N (cohort baseline)=30,239 | Non-severe | **Convenience**  Q1  Q2  Q3  Q4  **Plant-based**  Q1  Q2  Q3  Q4  **Sweets/Fats**  Q1  Q2  Q3  Q4  **Southern**  Q1  Q2  Q3  Q4  **Alcohol/salads**  Q1  Q2  Q3  Q4 | Ref 1.00  HRR=1.04 (0.86, 1.25)  HRR=0.97 (0.79, 1.17)  HRR=0.93 (0.74, 1.15)  Ref 1.00  HRR=1.22 (1.01, 1.47)  HRR=1.09 (0.89, 1.33)  HRR=0.89 (0.72, 1.12)  Ref 1.00  HRR=0.79 (0.65, 0.98)  HRR=0.91 (0.74, 1.11)  HRR=1.01 (0.81, 1.27)  Ref 1.00  HRR=1.20 (0.99, 1.47)  HRR=1.11 (0.89, 1.37)  HRR=1.39 (1.11, 1.73)  Ref 1.00  HRR=0.93 (0.77, 1.12)  HRR=0.91 (0.75, 1.10)  HRR=0.84 (0.69, 1.03) | Age, race, sex, geographic region of residence, and energy intake, waist circumference, annual income, educational achievement, physical activity, sedentary behavior, current  smoking, hypertension, diabetes, coronary artery disease, chronic pulmonary disease and chronic kidney disease | Acceptable quality |
| **Diabetes** | | | | | | | |
| Wang 2012[[36](#_ENREF_36)]  REGARDS-sepsis cohort study ^β^  2012-2015  The USA | Nationwide  Hospital ward, ED | Prospective cohort study analysis  N (cohort baseline)=30,239 | Non-severe | No  Yes | Ref 1.00  HRR=1.78 (1.53, 2.07) | age, sex, race, education, income, geographic region, alcohol use, and smoking status | Acceptable quality |
| Henriksen  2015b[[43](#_ENREF_43)]  Denmark | City/municipal  ED, ICU | Case-control study  N (cases)=1,713  N (controls)=227,054 | All | No  Yes | Ref 1.00  OR=1.82 (1.57, 2.12) | Age, alcoholism- related conditions, comorbidity, and immunosuppression | Low quality |
|  |  |  | Non-severe | No  Yes | Ref 1.00  OR=1.33 (1.01, 1.75) | See above |  |
|  |  |  | Severe | No  Yes | Ref 1.00  OR=2.02 (1.70, 2.41) | See above |  |
| **Immunosuppression** | | | | | | | |
| Henriksen  2015b[[43](#_ENREF_43)]  Denmark | City/municipal  ED, ICU | Case-control study  N (cases)=1,713  N (controls)=227,054 | All | No  Yes | Ref 1.00  OR=4.41 (3.83, 5.09) | Age, sex, alcoholism- related conditions, and comorbidity | Low quality |
|  |  |  | Non-severe | No  Yes | Ref 1.00  OR=5.03 (3.98, 6.34) |  |  |
|  |  |  | Severe | No  Yes | Ref 1.00  OR=4.45 (3.73, 5.30) |  |  |
| **Psychotic disorder** | | | | | | | |
| Henriksen  2015b[[43](#_ENREF_43)]  Denmark | City/municipal  ED, ICU | Case-control study  N (cases)=1,713  N (controls)=227,054 | All | No  Yes | Ref 1.00  OR=1.90 (1.58, 2.27) | Age, sex, alcoholism- related conditions, and comorbidity | Low quality |
|  |  |  | Non-severe | No  Yes | Ref 1.00  OR=1.35 (0.97, 1.88) |  |  |
|  |  |  | Severe | No  Yes | Ref 1.00  OR=2.93 (2.34, 3.67) |  |  |
| **Neurological disorder** | | | | | | | |
| Henriksen  2015b[[43](#_ENREF_43)]  Denmark | City/municipal  ED, ICU | Case-control study  N (cases)=1,713  N (controls)=227,054 | All | No  Yes | Ref 1.00  OR=1.98 (1.73, 2.26) | Age, sex, alcoholism- related conditions, and comorbidity | Low quality |
|  |  |  | Non-severe | No  Yes | Ref 1.00  OR=1.90 (1.52, 2.38) |  |  |
|  |  |  | Severe | No  Yes | Ref 1.00  OR=1.93 (1.65, 2.25) |  |  |
| **Chronic lung disease** | | | | | | | |
| Wang 2012[[36](#_ENREF_36)]  REGARDS-sepsis cohort study ^β^  2012-2015  The USA | Nationwide  Hospital ward, ED | Prospective cohort study analysis  N (cohort baseline)=30,239 | Non-severe | No  Yes | Ref 1.00  HRR=2.43 (2.05, 2.86) | Age, sex, race, education, income, geographic region, alcohol use, and smoking status | Acceptable quality |
| **Respiratory disorder** | | | | | | | |
| Henriksen  2015b[[43](#_ENREF_43)]  Denmark | City/municipal  ED, ICU | Case-control study  N (cases)=1,713  N (controls)=227,054 | All | No  Yes | Ref 1.00  OR=3.58 (3.16, 4.06) | Age, sex, alcoholism- related conditions, and comorbidity | Low quality |
|  |  |  | Non-severe | No  Yes | Ref 1.00  OR=3.70 (3.01, 4.54) |  |  |
|  |  |  | Severe | No  Yes | Ref 1.00  OR=3.29 (2.82, 3.84) |  |  |
| **Cancer** | | | | | | | |
| Henriksen  2015b[[43](#_ENREF_43)]  Denmark | City/municipal  ED, ICU | Case-control study  N (cases)=1,713  N (controls)=227,054 | All | No  Yes | Ref 1.00  OR=1.44 (1.22, 1.68) | Age, sex, alcoholism- related conditions, and comorbidity | Low quality |
|  |  |  | Non-severe | No  Yes | Ref 1.00  OR=1.30 (0.99, 1.72) |  |  |
|  |  |  | Severe | No  Yes | Ref 1.00  OR=1.47 (1.22, 1.78) |  |  |
| **Gastrointestinal disorder** | | | | | | | |
| Henriksen  2015b[[43](#_ENREF_43)]  Denmark | City/municipal  ED, ICU | Case-control study  N (cases)=1,713  N (controls)=227,054 | All | No  Yes | Ref 1.00  OR=1.71 (1.44, 2.05) | Age, sex, alcoholism- related conditions, and comorbidity | Low quality |
|  |  |  | Non-severe | No  Yes | Ref 1.00  OR=1.39 (1.02, 1.91) |  |  |
|  |  |  | Severe | No  Yes | Ref 1.00  OR=1.82 (1.48, 2.24) |  |  |
| **Chronic kidney disease** | | | | | | | |
| Wang 2012[[36](#_ENREF_36)]  REGARDS-sepsis cohort study ^β^  2012-2015  The USA | Nationwide  Hospital ward, ED | Prospective cohort study analysis  N (cohort baseline)=30,239 | Non-severe | No  Yes | Ref 1.00  HRR=1.99 (1.73, 2.29) | Age, sex, race, education, income, geographic region, alcohol use, and smoking status | Acceptable quality |
| **Renal disorder** | | | | | | | |
| Henriksen  2015b[[43](#_ENREF_43)]  Denmark | City/municipal  ED, ICU | Case-control study  N (cases)=1,713  N (controls)=227,054 | All | No  Yes | Ref 1.00  OR=1.46 (1.13, 1.89) | Age, sex, alcoholism- related conditions, and comorbidity | Low quality |
|  |  |  | Non-severe | No  Yes | Ref 1.00  OR=1.46 (0.95, 2.23) |  |  |
|  |  |  | Severe | No  Yes | Ref 1.00  OR=1.37 (1.01, 1.85) |  |  |
| **Peripheral artery disease** | | | | | | | |
| Wang 2012[[36](#_ENREF_36)]    REGARDS-sepsis cohort study ^β^  2012-2015  The USA | Nationwide  Hospital ward, ED | Prospective cohort study analysis  N (cohort baseline)=30,239 | Non-severe | No  Yes | Ref 1.00  HRR=2.16 (1.58, 2.95) | Age, sex, race, education, income, geographic region, alcohol use, and smoking status | Acceptable quality |
| **Myocardial infarction** | | | | | | | |
| Wang 2012[[36](#_ENREF_36)]  REGARDS-sepsis cohort study ^β^  2012-2015  The USA | Nationwide  Hospital ward, ED | Prospective cohort study analysis  N (cohort baseline)=30,239 | Non-severe | No  Yes | Ref 1.00  HRR=1.79 (1.49, 2.15) | Age, sex, race, education, income, geographic region, alcohol use, and smoking status | Acceptable quality |
| **Cardiovascular disease** | | | | | | | |
| Henriksen  2015b[[43](#_ENREF_43)]  Denmark | City/municipal  ED, ICU | Case-control study  N (cases)=1,713  N (controls)=227,054 | All | No  Yes | Ref 1.00  OR=1.62 (1.41, 1.85) | Age, sex, alcoholism- related conditions, and comorbidity | Low quality |
|  |  |  | Non-severe | No  Yes | Ref 1.00  OR=1.46 (1.15, 1.86) |  |  |
|  |  |  | Severe | No  Yes | Ref 1.00  OR=1.65 (1.40, 1.94) |  |  |
| **Atrial fibrillation** | | | | | | | |
| Wang 2012[[36](#_ENREF_36)]  REGARDS-sepsis cohort study ^β^  2012-2015  The USA | Nationwide  Hospital ward, ED | Prospective cohort study analysis  N (cohort baseline)=30,239 | Non-severe | No  Yes | Ref 1.00  HRR=1.48 (1.21, 1.81) | Age, sex, race, body mass index, education, income, geographic region, alcohol use, and smoking status | Acceptable quality |
| **Coronary artery disease** | | | | | | | |
| Wang 2012[[36](#_ENREF_36)]  REGARDS-sepsis cohort study ^β^  2012-2015  The USA | Nationwide  Hospital ward, ED | Prospective cohort study analysis  N (cohort baseline)=30,239 | Non-severe | No  Yes | Ref 1.00  HRR=1.61 (1.38, 1.87) | Age, sex, race, education, body mass index, race, income, geographic region, alcohol use, and smoking status | Acceptable quality |
| **Stroke** | | | | | | | |
| Wang 2012[[36](#_ENREF_36)]  REGARDS-sepsis cohort study ^β^  2012-2015  The USA | Nationwide  Hospital ward, ED | Prospective cohort study analysis  N (cohort baseline)=30,239 | Non-severe | No  Yes | Ref 1.00  HRR=1.67 (1.34, 2.07) | Age, sex, race, education, income, geographic region, alcohol use, and smoking status | Acceptable quality |
| **Deep vein thrombosis** | | | | | | | |
| Wang 2012[[36](#_ENREF_36)]  REGARDS-sepsis cohort study ^β^  2012-2015  The USA | Nationwide  Hospital ward, ED | Prospective cohort study analysis  N (cohort baseline)=30,239 | Non-severe | No  Yes | Ref 1.00  HRR=1.63 (1.29, 2.06) | Age, sex, race, body mass index, race, education, income, geographic region, alcohol use, and smoking status | Acceptable quality |
| **Hypertension** | | | | | | | |
| Wang 2012[[36](#_ENREF_36)]  REGARDS-sepsis cohort study ^β^  2012-2015  The USA | Nationwide  Hospital ward, ED | Prospective cohort study analysis  N (cohort baseline)=30,239 | Non-severe | No  Yes | Ref 1.00  HRR=1.49 (1.29, 1.74) | Age, sex, race, body mass index, education, income, geographic region, alcohol use, and smoking status | Acceptable quality |
| **Dyslipidaemia** | | | | | | | |
| Wang 2012[[36](#_ENREF_36)]  REGARDS-sepsis cohort study ^β^  2012-2015  The USA | Nationwide  Hospital ward, ED | Prospective cohort study analysis  N (cohort baseline)=30,239 | Non-severe | No  Yes | Ref 1.00  HRR=1.16 (1.01, 1.34) | Age, sex, race, body mass index, education, income, geographic region, alcohol use, and smoking status | Acceptable quality |
| **Serum 25 (OH) D levels (nmol/L)** | | | | | | | |
| Jovanovich 2014[[44](#_ENREF_44)]  The USA | Inter-State  Tertiary clinics, small clinics and hospitals | Case-control study  N (cases)=211  N (controls)=211 | Non-severe and severe sepsis | ≥50  <50 | Ref 1.00  OR=1.75 (1.11, 2.77) | Diabetes, renal disease, peripheral vascular disease | Low quality |
| **High sensitivity C-Reactive Protein (mg/L)** | | | | | | | |
| Wang 2012[[36](#_ENREF_36)]  REGARDS-sepsis cohort study ^β^  2012-2015  The USA | Nationwide  Hospital ward, ED | Prospective cohort study analysis  N (cohort baseline)=30,239 | Non-severe | ≤ 3.0  >3.0 | Ref 1.00  HRR=1.56 (1.36, 1.79) | Age, race, body mass index, race, sex, geographic region, income, education, smoking status, alcohol use, exercise, comorbidity | Acceptable quality |
| **Cystatin C (mg/dl)** | | | | | | | |
| Wang 2012[[36](#_ENREF_36)]  REGARDS-sepsis cohort study ^β^  2012-2015  The USA | Nationwide  Hospital ward, ED | Prospective cohort study analysis  N (cohort baseline)=30,239 | Non-severe | ≤ 1.12  >1.12 | Ref 1.00  HRR=1.51 (1.32, 1.72) | Age, sex, race, body mass index, geographic region, income, education, smoking status, alcohol use, exercise, comorbidity | Acceptable quality |
| **Endothelial and inflammation biomarkers (pg/mL or ng/mL)** | | | | | | | |
| Wang 2013c[[39](#_ENREF_39)]  REGARDS-sepsis case-control study  The USA | Nationwide  Hospital ward, ED | Case-control study  N (cases)=162  N (controls)=162 | Non-severe | **IL-6 (pg/mL)**  ≤1.92  >1.92-3.02  >3.02-4.73  >4.73  **TNF-α (pg/mL)**  ≤3.99  >3.99-5.19  >5.19-7.31  >7.31  **E-selectin (ng/mL)**  ≤34.52  >34.52-44.15  >44.15-56.22  >56.22  **ICAM-1 (ng/mL)**  ≤123.26  >123.26-149.56  >149.56-177.1  >177.1  **VCAM-1 (ng/mL)**  ≤1003.65  >1003.65-1173.66  >1173.66-1348.53  >1348.53 | Ref 1.00  OR=2.31 (1.01, 5.30)  OR=2.40 (1.11, 5.19)  OR=2.84 (1.26, 6.43)  Ref 1.00  OR=1.70 (0.82, 3.55)  OR=0.80 (0.38, 1.65)  OR=1.84 (0.93, 3.67)  Ref 1.00  OR=1.04 (0.53, 2.05)  OR=1.16 (0.57, 2.34)  OR=2.29 (1.11, 4.71)  Ref 1.00  OR=1.16 (0.56, 2.39)  OR=1.27 (0.64, 2.51)  OR=2.09 (1.05, 4.15)  Ref 1.00  OR=1.65 (0.79, 3.42)  OR=1.16 (0.58, 2.29)  OR=1.65 (0.80, 3.44) | Smoking, hypertension,  and CKD | Low quality |
| **Statin use** | | | | | | | |
| Wang 2012[[36](#_ENREF_36)]  REGARDS-sepsis cohort study ^β^  2012-2015  The USA | Nationwide  Hospital ward, ED | Prospective cohort study analysis  N (cohort baseline)=30,239 | Non-severe | No  Yes | Ref 1.00  HRR=0.93 (0.81, 1.06) | Patient demographics, health behaviours, chronic medical conditions, Morisky medication adherence, propensity for statin use | Acceptable quality |
| **NSAIDs/Aspirin use** | | | | | | | |
| Legras 2009 [[45](#_ENREF_45)] France | Regional  ICU | Case-control study  N (cases)=152  N (controls)=152 | Severe sepsis and septic shock | No  Yes | Ref 1.00  OR=0.93 (0.52, 1.64) | Not adjusted (crude) | Low quality |
| NR=not reported; ICU=intensive care unit; HDU=high dependence unit; ED=emergency department; 95% CI=95 percent confidence interval; REGARDS=Reasons for Geographic And Racial Differences in Stroke; Ref=reference group; OR=odds ratio; HRR=hazard rate ratio; NSAID=non-steroidal anti-inflammatory drug; CKD=chronic kidney disease; IL-6= interleukin-6; TNF-α=tumor necrosis factor alpha; ICAM=intercellular adhesion molecule; VCAM=vascular cell adhesion molecule; PSS=perceived stress scale; SD=standard deviation; Q1-4=dietary intake quartile scores | | | | | | | |

^β^ REGARDS-sepsis cohort study publications[[36](#_ENREF_36), [32-35](#_ENREF_32), [37](#_ENREF_37), [38](#_ENREF_38), [40](#_ENREF_40), [41](#_ENREF_41)]
